# Structural basis for conserved and distinct antigen recognition by a lineage of malaria-protective antibodies

**DOI:** 10.1371/journal.ppat.1014243

**Published:** 2026-06-03

**Authors:** Monika Jain, Fabien Cannac, Sashank Agrawal, Wen-Hsin Lee, Johannes R. Loeffler, Monica L. Fernández-Quintero, Gonzalo E. González-Páez, Re’em Moskovitz, Andrew B. Ward, Ian A. Wilson

**Affiliations:** 1 Department of Integrative Structural and Computational Biology, The Scripps Research Institute, La Jolla, California, United States of America; 2 The Skaggs Institute for Chemical Biology, The Scripps Research Institute, La Jolla, California, United States of America; University of Oxford, UNITED KINGDOM OF GREAT BRITAIN AND NORTHERN IRELAND

## Abstract

Monoclonal antibodies (mAbs) targeting the *Plasmodium falciparum* circumsporozoite protein (PfCSP) have demonstrated substantial promise in preventing malaria infection and disease. PfCSP is characterized by a central region composed of repetitive NANP motifs, which serve as major targets for protective antibodies. Several potent mAbs targeting this region exhibit homotypic Fab-Fab interactions, which enhance antigen binding and contribute to their neutralization potency. Among these, mAb 399, encoded by the *IGHV3-49/IGKV2D-29* (V_H_3-49/V_K_2D-29*)* germline lineages, forms head-to-head inter-Fab contacts mediated primarily by germline-encoded residues. Here, we determined X-ray and cryo-EM structures of two additional Fabs, derived from the same germline lineages, 7160 and 7118, in their unliganded forms and with PfCSP-derived peptides or recombinant shortened CSP. Both Fabs bound NANP_6_ repeats with high affinity (K_D_ 6–10 nM). Fab 7160 formed germline-encoded inter-Fab homotypic interactions resembling Fab 399, indicating a conserved and preconfigured mode of antigen recognition. In contrast, Fab 7118 does not form homotypic contacts and adopts a distinct binding mode, which precludes inter-Fab interactions. These findings highlight the structural versatility of V_H_3-49/V_K_2D-29-derived antibodies and demonstrate that their CDR loop variations can modulate antibody conformation, homotypic Fab-Fab interactions, and epitope engagement. Our study further defines this class of germline-encoded anti-CSP antibodies and provides mechanistic insights into how they achieve high-avidity binding and protective immunity either through or independent of pre-configured Fab-Fab interactions with important implications for germline-targeting malaria vaccine design.

## Author summary

Malaria vaccines targeting the *Plasmodium falciparum* circumsporozoite protein (CSP) provide partial protection that wanes over time, underscoring the need to better understand antibody-mediated immunity. In this study, we reveal how closely related human antibodies from the same germline lineage achieve potent protection through distinct structural mechanisms when binding the CSP repeat region. Using X-ray crystallography and cryo-electron microscopy, we demonstrate how subtle differences in antibody sequence, particularly within CDRH3, can determine whether antibodies form stabilizing inter-Fab homotypic interactions or instead engage CSP through alternative binding modes. These findings demonstrate structural adaptability within the V_H_3-49/V_K_2D-29-derived antibodies lineage to the CSP repeat region and provide molecular insights relevant for designing next-generation malaria vaccines that more effectively elicit durable and potent antibody responses.

## Introduction

Malaria remains a global concern and is endemic in many tropical countries, particularly in sub-Saharan Africa and Southeast Asia, despite substantial progress that has been made against malaria control over the past decades [1,2]. Recent reports indicate an increase in malaria cases, driven by factors such as climate change, insecticide-resistant mosquitoes, and drug-resistant parasites [3,4]. In 2024, an estimated 282 million cases and 610,000 deaths from malaria were reported worldwide [5]. Malaria is a mosquito-borne disease caused by unicellular parasites of the *Plasmodium* species. *Plasmodium falciparum* is the most lethal of these parasites, with a complex life cycle that involves both sexual and asexual stages occurring in humans and mosquitoes [6]. Several vaccines have been developed to target malaria, which are broadly categorized based on the *Plasmodium falciparum* life cycle: pre-erythrocytic vaccines, blood-stage vaccines, and transmission-blocking vaccines [7]. The circumsporozoite protein (CSP) of *P. falciparum* is the primary antigen targeted in the development of pre-erythrocytic vaccines. *Plasmodium falciparum* circumsporozoite protein (PfCSP) comprises three main regions: an N-terminal domain involved in hepatocyte attachment, a central immunodominant repeat region (comprising one NPDP, four NVDP, and approximately 35–41 NANP repeats), and a C-terminal thrombospondin repeat (αTSR) domain that mediates sporozoite attachment [8]. Among pre-erythrocytic vaccines, RTS,S and R21 are safe and effective in preventing malaria and have been approved by the World Health Organization (WHO) [9]. Both vaccines are based on a truncated version of CSP, containing 19 NANP repeats from the central region and the C-terminal αTSR domain. However, their efficacy declines over time [10–13]. One proposed explanation for this waning immunity is that the CSP repeat region may not effectively engage B cells, leading to the generation of predominantly short-lived plasmablasts rather than long-lived memory B cells [14,15].

**Fig 1:**
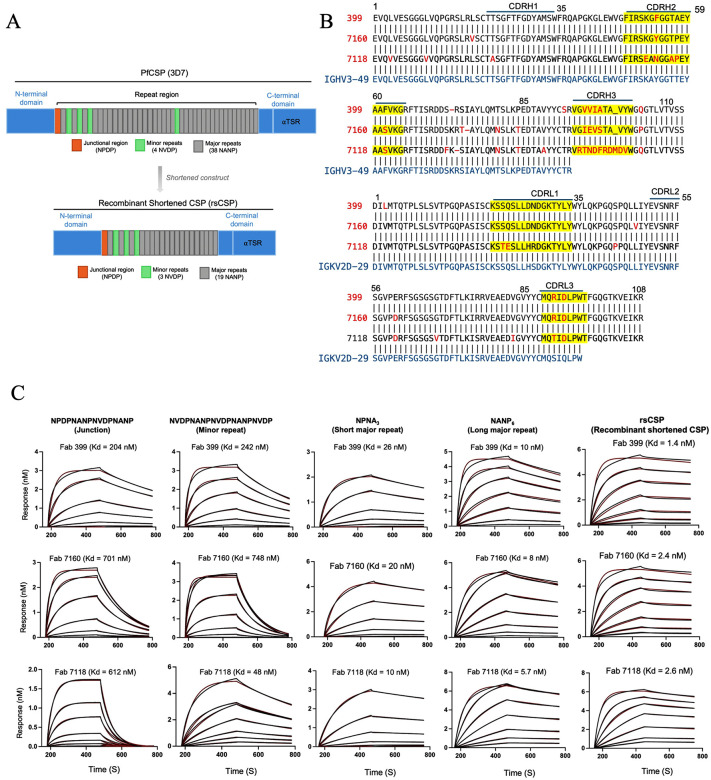
Domain organization of PfCSP and sequence and binding analysis of VH3-49/VK2D-29-derived antibodies. **(A)** PfCSP comprises an N-terminal domain (NTD), a central repeat region, and a C-terminal domain (CTD) containing a thrombospondin type-1 repeat (TSR). The central region consists mainly of NANP repeats interspersed with NVDP repeats and includes a junctional region between the NTD and the start of the NANP repeats. The rsCSP construct is a truncated recombinant form of PfCSP used for structural studies. **(B)** Sequence alignment of Fabs 7160, 7118, and 399 shows a shared VH3-49/VK2D-29 lineage. Amino acid differences are highlighted in red, CDRs in yellow, and alignment to the germline sequence in blue to identify somatic mutations. **(C)** Bio-layer interferometry (BLI) was used to determine binding kinetics of Fabs 399, 7160, and 7118 to rsCSP and PfCSP-derived peptides. Experimental and fitted curves (1:1 global model) are shown in red and black, respectively. Fab concentrations were serially diluted from top to bottom. Apparent dissociation constants (K_D_) are indicated.

Over the past few years, several human monoclonal antibodies (mAbs) specific to PfCSP have been isolated and shown to be protective in preclinical models [16]. Notably, highly potent mAbs such as CIS43, L9, and MAM01, each targeting different regions of CSP, specifically the junctional, minor repeat, and central repeat regions, respectively, have completed Phase I clinical trials [17–20]. Several high-resolution structures of antigen-binding fragments (Fabs) of protective monoclonal antibodies (mAbs) in complex with different regions of PfCSP have been elucidated using X-ray crystallography and cryo-electron microscopy [21–23]. Notably, a subset of mAbs encoded by diverse germline genes, including antibodies 850, 1210, 1450, 311, and 399, form Fab-Fab homotypic contacts upon binding to the central NANP repeat region and exhibit potent neutralizing activity [24–27]. Moreover, V_H_3-33-derived mAbs adopt a range of extended spiral conformations when complexed with rsCSP, which is a recombinant shortened PfCSP construct containing a reduced number of NANP/NVDP repeats: 19/3 repeats instead of 38/4 for the *P. falciparum* 3D7 strain (Fig 1A). Homotypic interactions between adjacent Fabs help stabilize this extended spiral configuration, enhancing binding avidity and potentially contributing to their protective efficacy [28]. In contrast, mAb 399, derived from the V_H_3-49/V_K_2D-29 germline lineage, has been reported to bind *Plasmodium falciparum* circumsporozoite protein (PfCSP) via inter-Fab homotypic contacts in a head-to-head configuration [25]. Notably, in this case, these contacts are largely mediated by germline-encoded residues, suggesting that antibodies from this lineage may have an innate tendency to form Fab-Fab contacts in the presence of CSP repeat peptides even without somatic hypermutation.

To further investigate this lineage, we examined mAbs 7160 and 7118, isolated from B-cells of protected individuals who participated in a Phase 2a clinical trial of the RTS,S/AS01 malaria vaccine [20,29]. These mAbs, 7160 and 7118, are both derived from V_H_3-49/V_K_2D-29 germline genes, showed high-affinity binding to the central NANP repeat region of PfCSP, and exhibited strong inhibitory activity, reducing liver-stage sporozoite burden by 90–95% in a murine challenge model. Notably, 7118 was isolated from a different vaccinee, suggesting that antibodies within this lineage can arise independently across individuals [20]. Sequence alignment with the previously characterized mAb 399 revealed high similarity across all three antibodies. However, mAb 7118 has a slightly different CDRH2 and a distinct CDRH3, which is extended by one additional amino acid residue relative to 399 and 7160 (Fig 1B).

To gain structural and mechanistic insights into this lineage, we determined high-resolution X-ray crystal structures of Fabs 7160 and 7118 in the presence or absence of PfCSP-derived peptides, as well as cryo-EM structures of Fabs 7160 and 7118 bound to rsCSP. Structural analysis revealed that Fab 7160 forms inter-Fab homotypic contacts both in the absence and presence of CSP-repeat peptides, closely resembling the self-association observed for unliganded Fab 399 in this study and for Fab 399 previously characterized in complex with NANP repeats [25]. In contrast, Fab 7118 did not display such interactions. Instead, its longer CDRH3 loop appears to create steric hindrance and electrostatic repulsion, preventing homotypic Fab-Fab contacts. Additionally, the extended CDRH3 induces a bend in the bound peptide, further differentiating its binding mode from that of 7160 and 399. These findings suggest that even within the same germline lineage, subtle differences in antibody sequence and structure can influence the formation of homotypic Fab-Fab interactions and interaction with the peptide. Together, our results highlight the structural adaptability of V_H_3-49/V_K_2D-29-derived antibodies and provide new molecular insights into how CSP-specific antibodies achieve high avidity and strong inhibition through distinct mechanistic strategies. Whereas Fab 7160 utilizes homotypic Fab-Fab interactions to promote multivalent binding, Fab 7118 relies on an alternative mode driven by its extended CDRH3, which precludes self-association. Collectively, these observations underscore how subtle sequence variations within a shared germline lineage can diversify antigen-binding mechanisms while preserving functional potency against CSP.

## Results

### Binding affinity of V_H_3-49/V_K_2D-29-derived potent mAbs 399, 7160, and 7118 to PfCSP-derived peptides and recombinant shortened CSP (rsCSP)

The antibody sequences of 7160 and 7118, as well as 399, were derived from plasmablasts isolated from protected individuals enrolled in a Phase 2a clinical trial of the RTS,S/AS01 vaccine, and both antibodies demonstrated protective activity in murine challenge models [20]. Fabs 7160, 7118, and 399 were recombinantly expressed and purified from a mammalian expression system, and peptides were synthesized for the binding and structure studies (see Methods and materials).

To evaluate the binding specificities of Fabs 7160, 7118, and 399 from the same V_H_3-49/V_K_2D-29 germline lineage, we performed Bio-layer Interferometry (BLI) using biotinylated peptides representing the junctional region (NPDPNANPNVDPNANP), minor repeat region (NVDPNANPNVDPNANPNVDP), and major repeats of varying lengths (NPNA₃ and NANP₆). All three Fabs bound the major repeats with high affinity, exhibiting dissociation constants (K_D_) in the range of 10–30 nM for NPNA₃ and 6–10 nM for NANP₆. Among them, Fab 7118 exhibited stronger binding to both major repeat peptides, with the lowest K_D_ values. Despite their preference for the major repeats, all Fabs also exhibited cross-reactivity with minor and junctional repeat peptides, but with reduced K_D_’s in the 50–750 nM range. Fab 399 showed approximately 9-fold lower affinity for both junctional and minor repeat peptides compared to NPNA_3_, whereas Fab 7160 exhibited ~20-fold lower affinity for these regions. Notably, Fab 7118 retained relatively strong binding to the minor repeat (K_D_ = 48 nM) yet displayed much weaker affinity for the junctional region (K_D_ = 612 nM), similar to the other antibodies (Fig 1C). These results indicate that these V_H_3-49/V_K_2D-29-derived antibodies preferentially bind the major repeat region of PfCSP while exhibiting some cross-reactivity to both junctional and minor repeats. To further assess binding in a more native-like context, we measured affinities to rsCSP. Biotinylated rsCSP was immobilized on streptavidin biosensors, and all Fabs exhibited higher affinity for rsCSP than for isolated repeat peptides, with K_D_ values ranging from 1.4 to 2.6 nM, indicating enhanced binding to rsCSP (Fig 1C and S1 Table).

### Crystal structures of Fab 7160 in complex with junctional, minor, major repeat peptides

**Fig 2:**
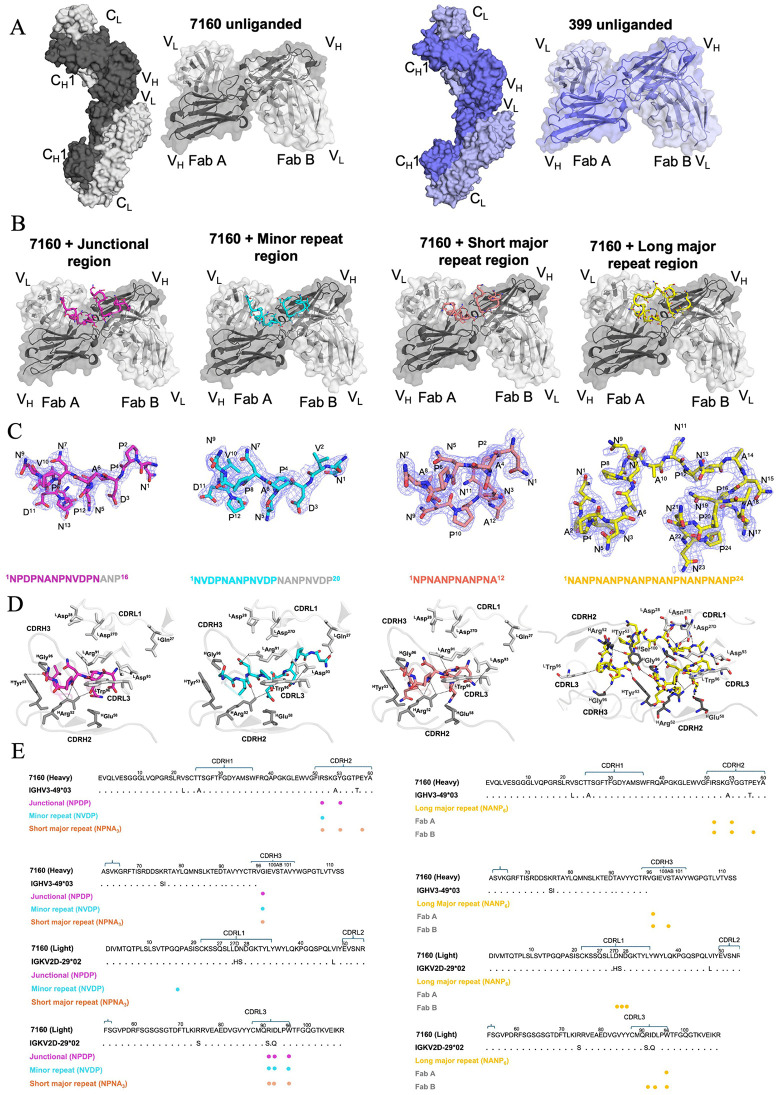
Structural basis of 7160 Fab binding to PfCSP peptides. **(A)** Surface and cartoon representations of unliganded Fab 7160 dimer and unliganded Fab 399 dimer, revealing homotypic head-to-head interactions. Fab A and B is colored dark grey (heavy chain) and light grey (light chain) for Fab 7160; Fab A and B is colored dark blue (heavy chain) and light blue (light chain) for Fab 399. **(B)** Surface representation of the 7160 Fab in complex with peptides from the junctional (magenta), minor repeat (cyan), short major repeat (salmon), and long major repeat (yellow) regions of PfCSP. The variable domains are colored dark grey (heavy chain) and light grey (light chain). **(C)** 2Fo–Fc electron density maps (contoured at 2.0σ, shown in grey) for each peptide bound to 7160: junctional (magenta), minor repeat (cyan), short major repeat (salmon), and long major repeat (yellow). Peptides are represented as sticks. **(D)** Close-up views of one Fab 7160-CDR interacting with junctional, minor, and short major repeat peptides, and two Fabs of 7160-CDRs interacting with long major repeat, are shown in stick representation and colored as in **(A)**. Hydrogen-bonding interactions between 7160 and each peptide are indicated by black dashed lines. **(E)** Fab 7160 amino acid sequence (Kabat numbering), with CDR residues involved in peptide binding marked by closed colored circles corresponding to each peptide region. The left panel shows residues involved in binding the junctional, minor repeat, and short major repeat peptides, whereas the right panel highlights residues involved in recognition of the long major repeat peptide, where two Fabs simultaneously engage the NANP_6_ peptide.

To gain mechanistic insights into these V_H_3-49/V_K_2D-29 mAbs, we determined unliganded crystal structures of Fab 399 and 7160 along with co-crystal structures of Fab 7160 in complex with PfCSP-derived peptides: junctional region, minor repeat region, short major repeat (NPNA₃), and long major central repeat (NANP₆) (Fig 2). The crystals of unliganded Fabs 399 and 7160 diffracted to resolutions of 2.29 Å and 2.39 Å, respectively (S2 Table), while the Fab-peptide co-crystals diffracted to resolutions of 2.30 Å, 2.27 Å, 1.90 Å, and 2.35 Å, respectively (S3 Table). All of the unliganded Fabs and Fab-peptide complexes assembled as dimers within the crystal lattice (Figs 2A, 2B, and S1A). The interactions between Fab 7160 and the peptides are primarily mediated by the heavy chain complementarity-determining regions CDRH2 and CDRL3, with additional contacts contributed by CDRH3 and, to a lesser extent, CDRL1 (S1B Fig).

In the Fab 7160-junctional peptide complex (^1^NPDPNANPNVDPNANP^16^), residues were numbered from 1 to 16. Electron density was present for residues 1–13 but absent for the last three C-terminal residues. In the complex with the minor repeat peptide (^1^NVDPNANPNVDPNANPNVDP^20^), residues were similarly numbered from 1 to 20, but no electron density was observed for Asn^13^ to Pro^20^. However, complexes of Fab 7160 with the short major repeat (^1^NPNANPNANPNA^12^) and long major repeat (^1^NANPNANPNANPNANPNANPNANP^24^) exhibited well-defined electron density for all peptide residues (Fig 2C). Fab 7160 recognizes all peptides in a similar conformation characterized by alternating type I β-turns and Asn pseudo-3₁₀ turns (S2 Fig). The core of the major repeat region adopts an almost identical conformation to that of the junctional region, with conserved hydrogen bond interactions involving heavy chain ^H^Arg^52^, ^H^Tyr^53^, ^H^Gly^96^, and light chain ^L^Arg^91^, ^L^Asp^93^, and ^L^Trp^96^ (Fig 2D). The 7160 Fab is bound to the minor repeat peptide with fewer hydrogen bonds in CDRH2, but with an additional hydrogen bond involving ^L^Gln^27^ in CDRL1. Consistent with the binding K_D_’s, Fab 7160 forms more extensive hydrogen bonding with the NANP_6_ peptide, and two Fab molecules simultaneously engage the long major repeat epitope through Fab-Fab homotypic interactions (Fig 2D and 2E).

### Fab 7160 recognizes short and long NANP repeat epitopes through a conserved binding mode shared with Fab 399

**Fig 3:**
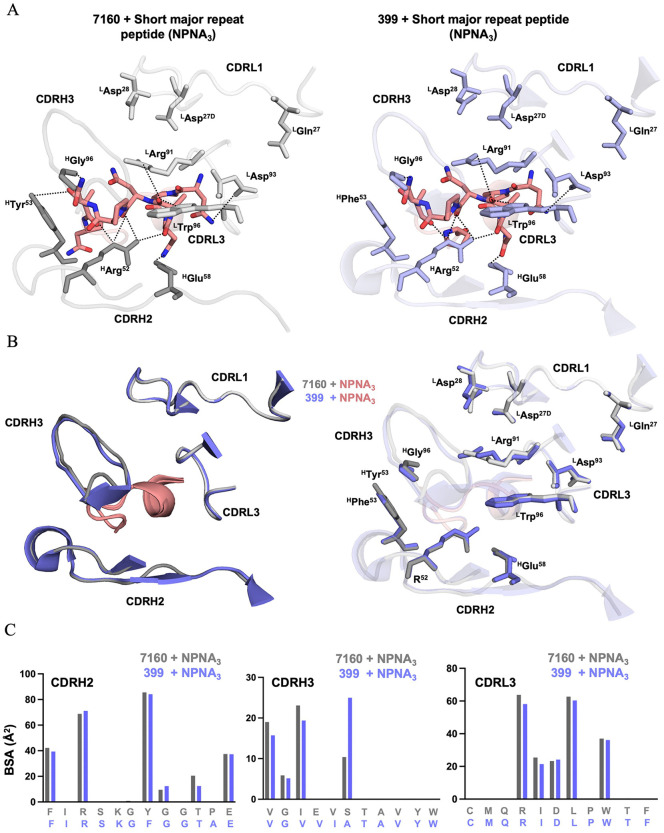
Conserved binding interactions between Fab 7160 and Fab 399. **(A)** Left: Crystal structure of 7160 in complex with NPNA_3_ peptide. Right: Crystal structure of Fab 399 bound to NPNA_3_ (PDB ID: 6WFZ). Only CDRs involved in peptide interactions are shown. Residues forming hydrogen bonds with the peptides are shown as sticks. **(B)** Structural alignment of Fabs 399 (blue) and 7160 (grey) in complex with NPNA_3_ (salmon). **(C)** Bar plot depicting buried surface area contributions from CDRs H2, H3, and L3 in Fab 399 (blue) and 7160 (grey) with bound NPNA_3_ peptide.

To further analyze the epitope recognition by Fab 7160, we compared the crystal structures of Fab 7160 in complex with short and long peptides derived from the PfCSP central NANP repeat region to the previously reported structures of Fab 399 [25] (Fig 3A). Superimposition of the variable domains of the 7160-NPNA₃ and 399-NPNA₃ complexes revealed a root-mean-square deviation (RMSD) of only 0.26 Å across all C-alpha atoms, indicating a nearly identical overall conformation (Fig 3B). Despite the high structural similarity, Fab 7160 possesses a germline-encoded ^H^Tyr^53^ in CDRH2 that forms additional contacts with the epitope, whereas Fab 399 has ^H^Phe^53^ in the same position. Furthermore, the buried surface area (BSA) in the Fab 7160-NPNA₃ complex contributed by the heavy chain CDRH2 and CDRH3 (310 Å² on the Fab and 363 Å² on the peptide) is similar to that of the Fab 399-NPNA₃ complex (308 Å² on the Fab and 358 Å² on the peptide). In both complexes, the light chain contribution is also substantial and mediated primarily by CDRL3, with the Fab 7160 light chain having a BSA of 215 Å² and 214 Å² on the peptide, comparable to Fab 399 (201 Å² on the Fab and 200 Å² on the peptide) (Fig 3C). These findings demonstrate that Fab 7160 engages NPNA₃ in a nearly identical manner to Fab 399, suggesting conserved recognition features for NANP repeats within this lineage.

**Fig 4:**
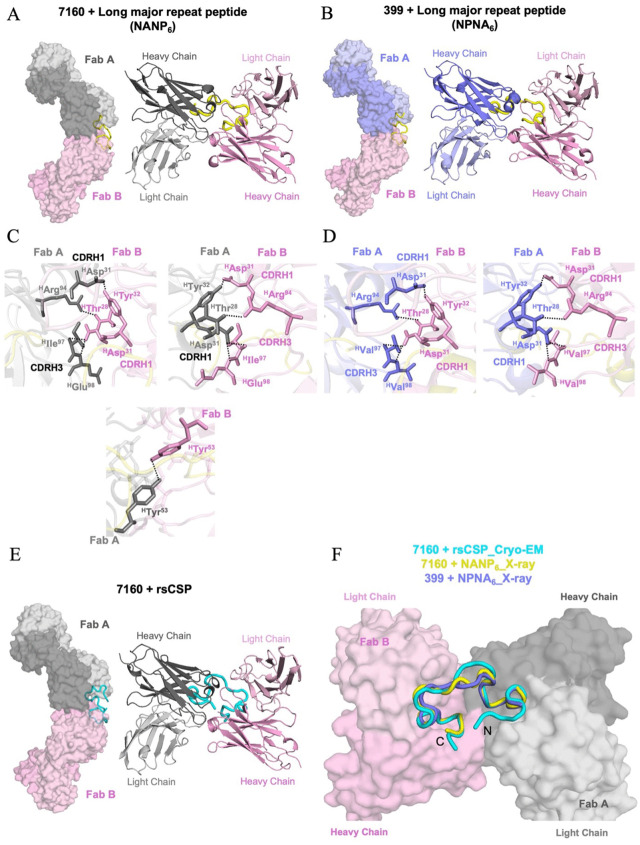
Homotypic Fab-Fab interactions and peptide engagement by Fabs 7160 and 399. **(A)** Homotypic head-to-head interactions between two Fab 7160 molecules are shown in both surface and cartoon representations. Fab A is colored dark grey (heavy chain) and light grey (light chain); Fab B is shown in dark pink (heavy chain) and light pink (light chain). The NANP_6_ peptide is depicted in a yellow cartoon. **(B)** Crystal structure of two Fab 399 molecules bound to NANP_6_ (PDB ID: 6WG1), displayed as surfaces and in cartoon form. Fab A is colored dark blue (heavy chain) and light blue (light chain), and Fab B is colored dark pink (heavy chain) and light pink (light chain). **(C)** Hydrogen-bonding interactions within the homotypic interface formed by two Fab 7160 molecules simultaneously engaging the NANP_6_ peptide. **(D)** Interactions between the two Fab 399 molecules simultaneously engage an NPNA_6_ peptide. Hydrogen bonds are indicated by black dashed lines. The Fabs are shown as cartoon representations with the side chains of interacting residues represented as sticks. **(E)** Cryo-EM structure of Fab 7160 in complex with rsCSP. Two Fab 7160 molecules bound to rsCSP are shown in surface and cartoon representations, with rsCSP colored cyan. **(F)** Structural overlay comparing the cryo-EM structure of 7160-rsCSP (cyan) with the X-ray structures of Fab 7160 (yellow) and 399 (dark blue) bound to CSP-derived NANP_6_ peptide.

Next, we compared the crystal structure of Fab 7160 in complex with the long major repeat region peptide (NANP₆) to that of the previously characterized Fab 399-NANP₆ complex (PDB ID: 6WG1) [25] (Fig 4A and 4B). Fab 7160 exhibited slightly higher binding affinity and a modestly increased number of hydrogen bonds and inter-Fab contacts with NANP₆ compared to Fab 399 (S3 Fig). The Fab-Fab interactions in both Fab 399 and Fab 7160 complexes are symmetric, with CDRH1 and CDRH3 of one Fab engaging CDRH1 of the adjacent Fab and vice-versa (Fig 4C). Specifically, ^H^Asp^31^ in CDRH1 and ^H^Arg^94^ in CDRH3 form hydrogen bonds with ^H^Tyr^32^ and ^H^Thr^28^, respectively, in the neighboring Fab.

Additionally, ^H^Ile^97^ and ^H^Glu^98^ of Fab 7160 form a network of mainchain-to-sidechain hydrogen bonds with ^H^Asp^31^ of the adjacent Fab. A similar hydrogen-bonding pattern was observed in the Fab 399-NANP_6_ complex (Fig 4D) [25]. Importantly, ^H^Tyr^53^ in Fab 7160 plays a dual role by mediating key homotypic Fab-Fab contacts and directly participating in CSP binding (Figs 4C and 3A). Overall, Fab 7160 adopts a mode of recognition nearly identical to that of Fab 399 for both short and long NANP repeat epitopes, characterized by conserved epitope contacts and symmetric, head-to-head Fab-Fab interactions. This binding configuration represents a unique binding strategy among PfCSP-directed antibodies, as the formation of homotypic contacts is symmetric, dominated by the heavy chain, and is largely independent of somatic hypermutation (SHM) [25]. This contrasts with the spiral arrangement adopted by antibodies derived from the V_H_3-33 germline that are predominantly expressed among PfCSP-binding antibodies, highlighting diverse lineage-specific modes of antigen recognition.

### Cryo-EM of Fab 7160 in complex with recombinant rsCSP

Both the previously reported Fab 399-NANP_6_ and our new Fab 7160-NANP_6_ crystal structures display a head-to-head interface with a relatively short peptide bound in between the two Fab molecules, posing the question of whether a longer antigen, such as CSP, would accommodate such a tight interface. To assess whether the Fab 7160 interface allows a CSP antigen to fully occupy the binding sites on both Fab molecules simultaneously, we solved a cryo-EM structure of Fab 7160 in complex with rsCSP, as previously described [28]. The cryo-EM structure at 3.55 Å resolution had two Fab molecules clearly visible bound to rsCSP (S4 Fig). Both X-ray and cryo-EM structures of the Fab dimers are nearly identical, with an overall RMSD of 0.47 Å (S5A Fig). In the cryo-EM structure, however, both epitopes are fully occupied by rsCSP with density for 31 antigen residues (vs. 24 for the crystal structure), confirming that the head-to-head interface of the Fab variable domains does not hinder binding to the CSP antigen. As expected from the symmetrical Fab-Fab interface, antigen binding is also relatively symmetrical (Fig 4E), with the N and C-termini of the bound NANP repeats in the rsCSP exiting the complex on the same side. The short linker region between the CSP epitopes on adjacent Fabs is flexible and adopts different conformation in the crystal and cryo-EM structures (Figs 4F and S5A), but enables each epitope to orient correctly for Fab binding on either side. Furthermore, although we could not obtain 3D models, we observed 2D classes indicating a higher-order arrangement of the Fabs with oligomerization of the dimers (S5B Fig). Attempts at reconstructing 3D maps and models from these particles were, however, unsuccessful, as the lack of alternate views led to orientation bias. A 3D map shows distinct density that can be attributed to four 7160 Fab molecules (S5B Fig) but lacked the clarity to build high-resolution models. Nonetheless, these are clear indications that multiple Fab-7160 complexes can coexist on one CSP antigen, and more investigation will be required to solve their 3D cryo-EM structures.

This cryo-EM study represents the first complex structure of an antibody from this lineage with a longer CSP molecule and confirms that, despite the tight space at the Fab-Fab interface, such antibodies are capable of binding the antigen as it would be presented on the parasite, while retaining their homodimeric form and with multiple antibodies binding the same antigen.

### Crystal structures of Fab 7118 reveal a unique binding mode for the PfCSP repeat region

**Fig 5:**
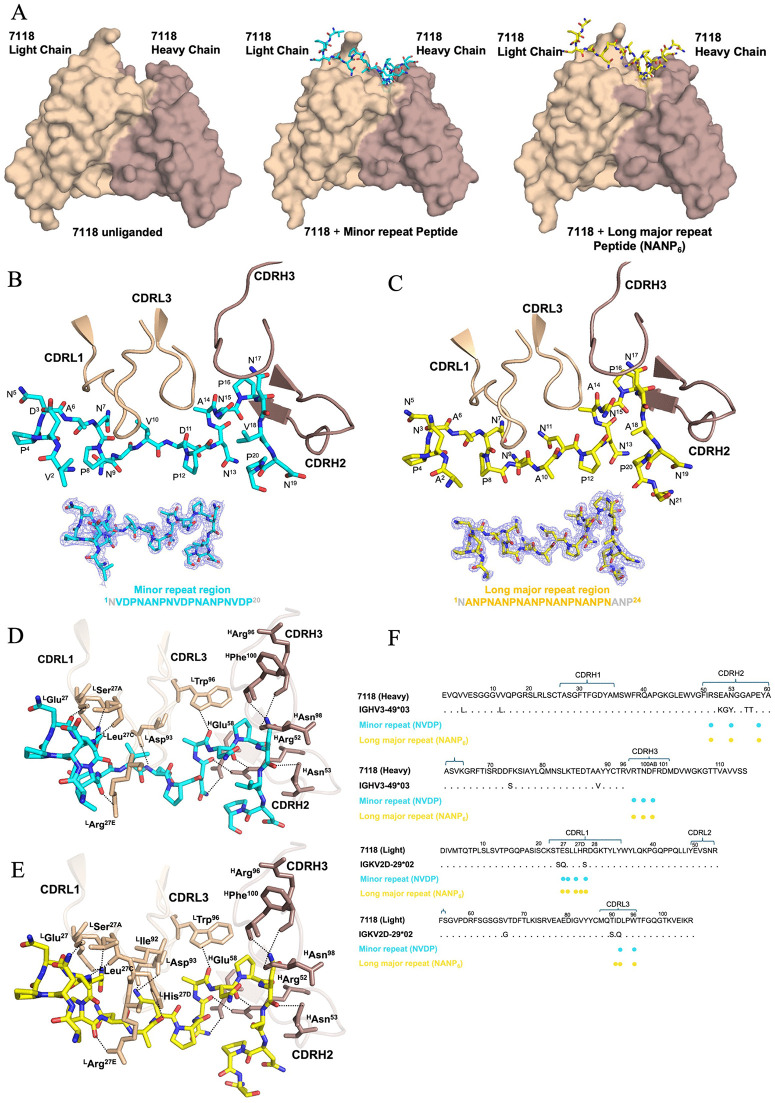
Structural basis of 7118 Fab binding to minor and major repeat peptides. **(A)** Surface representation of the unliganded Fab 7118 and in complex with peptides from the minor repeat (cyan) and long major repeat (yellow) regions of PfCSP. The variable domains are colored brown (heavy chain) and wheat (light chain). **(B-C)** Close-up views of 7118-CDRs interacting with each peptide, shown as stick representation and colored as in panels A and B. 2Fo–Fc electron density maps (contoured at 2.0σ, shown in grey) for each peptide bound to 7118: minor repeat (cyan), and long major repeat (yellow). Peptides are represented as sticks. **(D-E)** Details of hydrogen-bonding interactions between 7118 and each peptide are shown as black dashed lines. **(F)** Amino acid sequence of Fab 7118 (Kabat numbering), with CDR residues involved in peptide binding marked by closed colored circles corresponding to the peptide region.

To investigate the structural basis of Fab 7118 recognition of the PfCSP, we determined X-ray structures of Fab 7118 in the absence or presence of CSP peptides derived from the minor repeat region and long major central repeat region of PfCSP. The crystals of unliganded Fab and Fab-peptide complexes diffracted to high resolutions of 2.29 Å, 2.09 Å, and 2.00 Å, respectively (Fig 5A, and S2 and S4 Tables). The interactions between Fab 7118 and peptides are mediated by CDRH2, CDRH3, CDRL1, and CDRL3 (Fig 5B). In the 7118 Fab complex with the minor repeat peptide (^1^NVDPNANPNVDPNANPNVDP^20^), the electron density for Asn^1^ was poorly defined. In the complex with long major repeat (^1^NANPNANPNANPNANPNANPNANP^24^), electron density was absent for the first N-terminal and last three C-terminal residues (Fig 5B and 5C). The core of the major repeat region adopts an almost identical conformation to that of the minor repeat region (Fig 5D and 5E). The conserved hydrogen bond interactions to the peptides involve heavy chain ^H^Arg^52^, ^H^Asn^53^, ^H^Glu^58^, ^H^Arg^96^, ^H^Asn^98^ and ^H^Phe^100^, and light chain ^L^Glu^27^, ^L^Ser^27A^, ^L^Leu^27C^, ^L^Arg^27E^, ^L^Asp^93^, and ^L^Trp^96^ (Fig 5F). Unlike Fabs 399 and 7160, Fab 7118 does not form inter-Fab homotypic interactions. Instead, 7118 engages in extensive hydrogen bonding with its peptide epitope. In particular, CDRH3 and CDRL1 of 7118 form an additional and distinct set of hydrogen bond contacts compared to those observed in 399 or 7160 (Tables A and B in S5 Table). These additional interactions in Fab 7118 likely compensate for the absence of inter-Fab stabilization, helping to maintain high-affinity binding. Structural superimposition of Fab 7118 and Fab 7160 in complex with NANP_6_ highlights a distinct peptide-binding mode in 7118, where all interactions with the peptide are accommodated within a single Fab (S6 Fig). Notably, the CDRH3 loop in 7118 is longer and contains charged (Arg, Asp) as well as bulky (Phe) residues (S6A Fig). These features likely introduce steric hindrance and electrostatic repulsion, thereby preventing head-to-head Fab-Fab contacts observed in 7160 and 399 (S6B and S6C Fig). This extended CDRH3 induces a bend in the bound peptide (S6D Fig), suggesting an alternative mechanism for achieving affinity and stabilizing the Fab-epitope complex in the absence of homotypic interactions. Nevertheless, despite these differences in peptide-binding mode, all three Fabs recognize a conserved and similar binding mode for two NANP repeats.

### Cryo-EM and molecular dynamics simulations of Fab 7118 bound to rsCSP demonstrate a quaternary spiral structure with transient homotypic contacts

**Fig 6:**
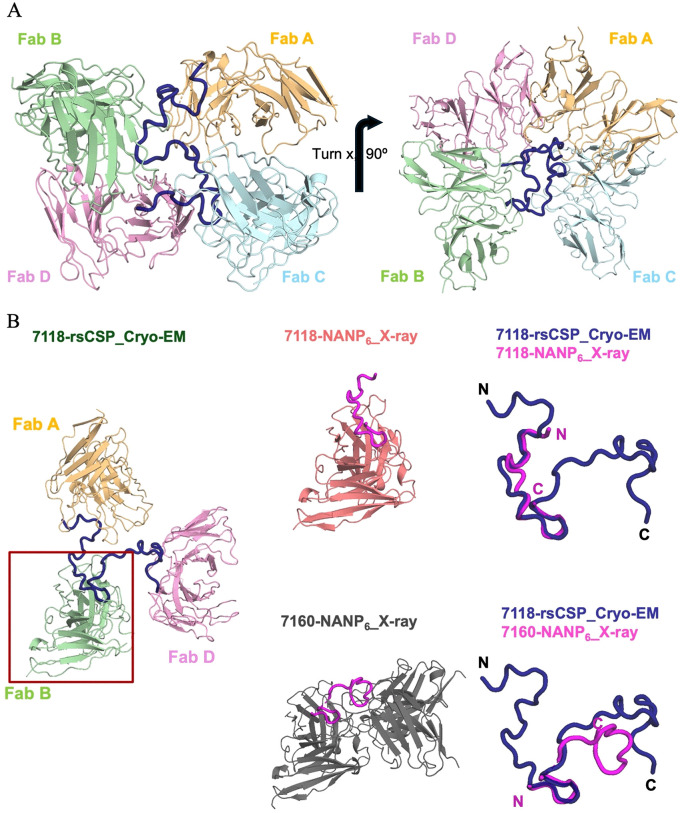
Cryo-EM structure of the 7118 Fab-rsCSP complex. **(A)** Cryo-EM structure of 7118-rsCSP complex at 3.32 Å resolution. A cartoon representation of the atomic model is shown; only the Fab variable regions (Fv) were built into the density. Individual Fabs are colored (Fab A: wheat, Fab B: green, Fab C: cyan, Fab D: pink) and rsCSP is represented as a blue backbone tube. The right panel shows a rotated view. **(B)** Comparison and superposition of Fab variable regions from the 7118-rsCSP cryo-EM structure (green; only Fabs A, B, and D shown) with the 7118-NANP_6_ crystal structure (salmon, upper right) and two Fab molecules from the 7160-NANP_6_ crystal structure (grey, lower right), highlighting similarities and differences in the peptide binding modes. Models were aligned in PyMOL [30] and displayed in cartoon representation to highlight variations in how the Fabs recognize the NANP repeats.

To determine how Fab 7118 binds to CSP, we determined a cryo-EM structure of Fab 7118 in complex with rsCSP. The cryo-EM map was resolved to an overall resolution of 3.32 Å (S7 Fig and S6 Table), allowing visualization of the Fab variable domains bound to repetitive NANP motifs within the central repeat region of rsCSP (Fig 6A). The structure revealed that four Fab molecules simultaneously bind to the repeating epitopes within rsCSP but without engaging in direct inter-Fab homotypic contacts. Structural superposition of the cryo-EM model with the Fab 7118-peptide crystal structure showed minimal conformational differences in the Fab variable domain and bound peptide. This structure contrasts with Fab 7160, which adopts a different peptide-binding orientation in its crystal structure and exhibits inter-Fab contacts. Despite these differences, two NANP repeats are recognized identically across these Fabs, adopting a similar configuration (Fig 6B).

When the Fab 7118 cryo-EM structure was superimposed onto the Fab 7160-NANP_6_ complex, we observed steric clashes at potential inter-Fab contact sites, indicating structural incompatibility. These clashes are primarily localized to the longer CDRH3 loop of Fab 7118, particularly at ^H^Asp^99^, which likely introduces steric hindrance that prevents homotypic Fab-Fab interactions and is predicted to interfere with the Fab-Fab packing seen in 7160 and 399 (S8 Fig). Collectively, these findings highlight the unique binding configuration of Fab 7118, where additional engagement with the epitope compensates for the lack of inter-Fab interactions, enabling strong and specific recognition of the PfCSP repeat region.

While this cryo-EM binding mode is consistent with the crystal structure and confirms that Fab 7118 maintains a monovalent mode of engagement with each repeat epitope, we investigated the stability of the quaternary structure observed in the absence of strong homotypic contacts. We performed 3D Variability (3DVA) [31] on the cryo-EM data using particles from the final reconstruction. The resulting volumes described a continuous variation across the three different modes solved, with more pronounced variance in voxel value on the C_H_1 domain of the Fab molecules highlighting motion on the outside of the spiral (S9A Fig). This motion is occurring as individual Fab molecules pivot around a constrained CSP spiral and is homogeneous, as demonstrated by the even distribution of reaction coordinates (S9B Fig), suggesting a continuous motion, confirmed by a movie showing rocking of the Fab around the CSP helix (S1 Movie).

**Fig 7:**
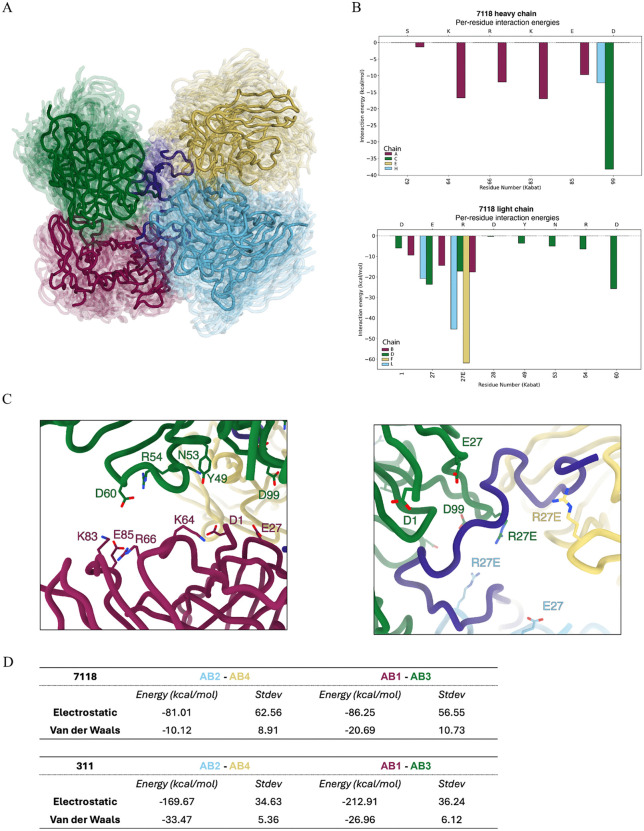
Molecular dynamics simulations of 7118-rsCSP complex. **(A)** Overlay of all clusters from the molecular dynamics simulations. All three 1 μs simulations were averaged into one trajectory, itself split into 40 clusters based on atom coordinate standard deviations. The most populated cluster is represented as a solid ribbon, and the other clusters as transparent ribbons. All four 7118 Fabs (1: maroon, 2: cyan, 3: green, 4: yellow) are represented as ribbons, rsCSP (blue) as sticks with heteroatoms colored (dark blue: nitrogen, red: oxygen). **(B)** Transient 7118 homotypic contacts observed in MD (color code as above), showing the averaged electrostatic energy of contact for each residue position and each Fab, following the color code above. **(C)** Residues from B) highlighted as stick figures on the deposited PDB model from the cryo-EM of 7118 – rsCSP. **(D)** Comparison of Fab-Fab energy interface between 7118 and 311 cryo-EM models, averaging the calculated energy for each complex over the course of the molecular dynamics simulations.

To further characterize this motion and the interaction networks and energetics involved in spiral formation and stabilization, we performed molecular dynamics (MD) simulations on our cryo-EM model. The MD simulations revealed high flexibility of the complex reflected in a high conformational diversity, in line with the motions observed in 3DVA (Fig 7A). Next, we calculated the respective interaction energies between adjacent Fab molecules over the whole simulation trajectory; for each interacting residue, the average energy was calculated and the resulting value plotted (Fig 7B), revealing transient homotypic contacts between neighboring Fab molecules (Fig 7C). These interactions are short-lived, occurring for less than 20% of the total molecular dynamics simulation time (2 µs). Their transient nature makes it difficult to calculate an exact number for how long they last, as they could form for 20 ns and then dissipate, or form and break 10–20 times within these same 20 ns. Yet overall, none has a cumulative time of more than 20% of the 2 µs (so ~ 400 ns). Cumulated over the course of the three 1 µs simulations, none of these transient contacts occurred more than 20% of the time, highlighting how ephemeral they are. None of the interacting residues on the heavy chain is mutated from germline (all but one are on framework 3, with D99 on HCDR3). Similarly, on the light chain, all residues are also germline, except for R27E on LCDR1.

To define the strength of these homotypic contacts, we compared the average energy at the transient interface of three consecutive 7118 Fab molecules with three Fab molecules from a classic homotypic spiral, like 311 Fab (PDB: 8DYX, Fig 7D). Calculations indicate that the overall electrostatic energy for 311 Fab-Fab interfaces is more than twice that of 7118, with only half the standard deviation. A similar trend is observed for van der Waals energies, largely driven by hydrophobic residues, none of which are involved in transient interactions in 7118. While the energies of the homotypic interface interactions are relatively weak, the CSP-binding energies range from -120 and -139 kcal/mol for three of the antibodies, confirming the strong epitope/paratope interactions (S7 Table). The density at the C-terminal end of the spiral was too weak to confidently build the full peptide in the binding site, reflecting the weaker calculated binding in the simulation for this Fab. Altogether, these data indicate that while 7118 complexed with CSP forms a quaternary structure with a spiral conformation, it does not adopt the homotypic states described previously. Whereas the stability of a classic homotypic spiral, such as with antibody 311, is mediated through strong Fab-Fab electrostatic and van der Waals interactions [26,28], 7118 does not engage in stable homotypic interactions. Instead, any Fab-Fab interactions observed in simulations are transient and weak, and restricted to adjacent Fabs without simultaneous engagement of multiple neighboring Fabs. These observations suggest that 7118 may possibly represent a maturation intermediate of a CSP-binding antibody on the way to acquiring the somatic hypermutations necessary to form a more permanent homotypic state. Previous work has suggested that affinity maturation is a key component of homotypic formation [28]. We show that several mAb 7118 molecules are already able to bind to the CSP antigen in proximity to each other, a spatial prerequisite for the formation of homotypic contacts between neighboring antibodies. Affinity maturation of other residues could potentially stabilize a spiral architecture by reducing conformational flexibility and strengthening a homotypic state with more permanent contacts. It will therefore be interesting to identify and characterize additional antibodies from the V_H_3-49/V_K_2D-29 germline lineage to define the maturation pathways and somatic hypermutation patterns associated with homotypic Fab interactions and spiral formation.

### Germline-encoded interactions of aromatic Fabs 7160, 7118, and 399

**Fig 8:**
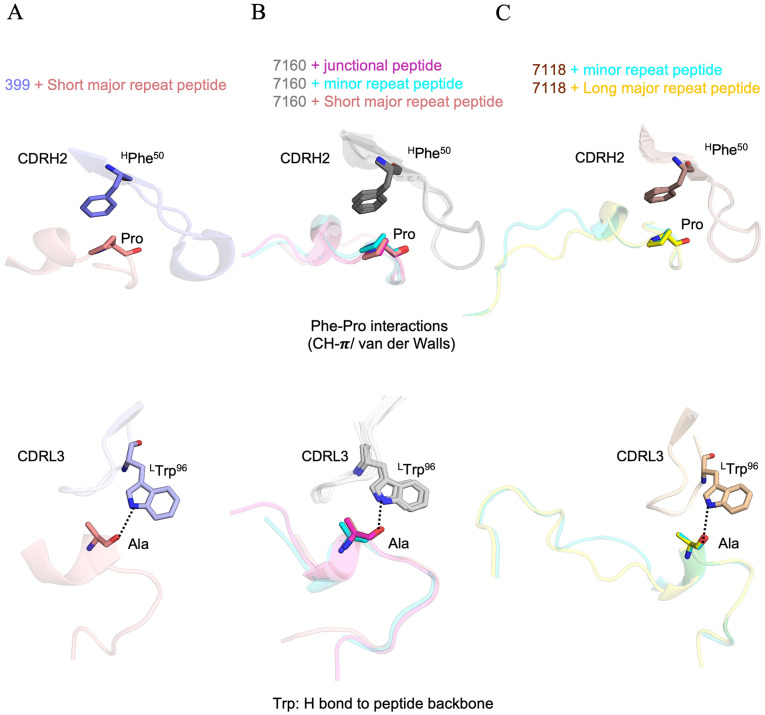
Interactions between Fab aromatic residues and CSP-derived peptides. **(A)** The Fab 399 aromatic residues (blue sticks) interacting with the NPNA_3_ peptide (salmon sticks). **(B)** Superimposed view of 7160 Fab aromatic residues (grey sticks) interacting with peptides from the junctional (magenta), minor repeat (cyan), and short major repeat region (salmon). **(C)** Superimposed view of Fab 7118 aromatic residues (brown sticks) in complex with peptides from minor repeat (cyan), and major repeat region (yellow). Hydrogen bonds are represented as black dashed lines. Residue identities and numbers are labeled with H and L, denoting heavy and light chain, respectively.

Structural comparisons of Fab 7160 and Fab 7118 bound to PfCSP-derived peptides revealed conserved aromatic interactions that were also present in the Fab 399-NPNA₃ complex [25]. In all three Fab-peptide complexes, a phenylalanine at position 50 in CDRH2, encoded by the *IGHV3–49* germline gene, forms CH/π interactions with the conserved proline residue within the NPNA motif. This interaction is further supported by a germline-encoded tryptophan at position 96 in CDRL3 (V_K_2D-29), which forms a hydrogen bond with the backbone carbonyl of the alanine residue preceding the type I β-turn (Fig 8). Notably, two NANP repeats in these Fabs bind identically, adopting a similar configuration and exhibiting conserved CH/π interactions with their epitopes. These features are consistently observed in the high-resolution crystal structures of 7160 and 7118 bound to peptides from the junctional, minor, and long major repeat regions of PfCSP. Together, these structural findings indicate that these V_H_3-49/V_K_2D-29 Fabs utilize a conserved set of aromatic and hydrogen-bonding interactions to recognize the PfCSP-derived epitopes across different regions.

## Discussion

Our structural characterization of PfCSP-specific mAbs derived from the V_H_3-49/V_K_2D-29 germline lineage provides insights into how antibodies from the same genetic origin can achieve high-affinity binding and potent protection through diverse binding strategies. To facilitate comparison of antibodies analyzed in this study, we summarized their genetic and clonal characteristics (S8 Table). Although antibodies 399, 7160, and 7118 are all derived from the same germline lineages, antibody 7118 originated from an independent B-cell clone, as indicated by a distinct CDRH3 sequence. The presence of clonally distinct antibodies within the same germline framework highlights the structural and clonal diversity that can arise within a single public antibody lineage.

Fab 7160, like Fab 399, forms inter-Fab homotypic contacts in a symmetric head-to-head configuration both in the absence and presence of CSP repeat peptides. These preconfigured Fab-Fab interactions appear to be germline-encoded. They are primarily mediated by heavy chain residues within CDRH1 and CDRH3, with contributions from CDRH2 and CDRL1, indicating that this lineage possesses an intrinsic propensity for self-association. The persistence of inter-Fab interactions independent of antigen engagement suggests that Fab 399 and Fab 7160 are structurally preorganized for cooperative binding to repetitive NANP motifs. In contrast, Fab 7118, which contains a slightly extended CDRH3 that disrupts Fab-Fab assembly, does not form homotypic interactions, yet exhibits comparable or even higher affinity for PfCSP repeat peptides and potent inhibition.

Although the antibodies examined here have been reported to confer protection in murine challenge models, the extent to which their distinct binding modes influence protective efficacy or mechanism remains unclear. One possibility is that homotypic Fab-Fab interactions stabilize specific conformations of CSP, facilitating cooperative engagement of neighboring Fabs, increasing the antibody density on the parasite surface, thereby enhancing the functional activity. Alternatively, antibodies such as 7118, which lack stable inter-Fab contacts, may rely on different mechanisms, such as increased interactions with antigen rather than avidity acquired from Fab-Fab homotypic interactions, to achieve protection. These observations suggest that multiple structural solutions can ultimately lead to effective immune protection. Also, our structural studies identified specific residues that mediate homotypic Fab-Fab interactions in antibodies 399 and 7160, providing a framework for future functional examination. Studies involving site-directed mutagenesis of interface residues and functional analyses in binding or protection assays would also help clarify the extent to which the homotypic contacts described here contribute to antibody potency in vivo. Such experiments will further inform us on whether Fab self-association represents a general mechanism for enhancing the efficacy of antibodies targeting the repetitive repeat sequences of PfCSP, and whether disruption of these interactions alters protective efficacy in vivo. This work expands our understanding of how germline-related antibodies engage the extensive repeat sequences in malaria antigens and highlights the importance of structural diversity in shaping effective humoral immune responses. Further integration of structural, biochemical, and in vivo studies will be essential to define the functional consequences of these distinct binding modes.

Moreover, structural analysis by Cryo-EM reveals that Fab 7118 binds in a spiral configuration without homotypic Fab-Fab contacts, although MD simulation suggests transient contacts can form between the Fab molecules that are not captured in the cryo-EM ensemble, possibly due to its transitory nature. The elongated CDRH3 loop introduces steric hindrance and potential electrostatic repulsion that also likely prevents stable inter-Fab contacts, while simultaneously inducing a bend in the bound NANP₆ peptide. These features were consistently observed in both crystal and cryo-EM structures of Fab 7118, confirming that its distinct binding mode is preserved in the context of the rsCSP antigen.

Molecular dynamics simulations to characterize the energy of Fab 7118-rsCSP quaternary structure further revealed transient homotypic contacts that are weaker than those of published homotypic spirals like Fab 311 [26,28]. Structurally, 7118 displays a hybrid phenotype between the previously characterized antibodies 311 and 399 or 7160, e.g., transient inter-Fab contacts, reduced cooperativity, or distinct binding geometry. These observations suggest that 7118 may represent an alternative, intermediate, or transitional pathway in the maturation of the IGHV3–49/IGKV2D-29 lineage, where the mode of PfCSP engagement resembles, but does not fully stabilize, the spiral architecture. Consistent with this behavior, we observe a maximum of ~4 Fab 7118 molecules bound to rsCSP, compared to the ~ 11 copies reported for Fab 311. Fab 311 engages the repetitive NANP region in a manner that supports dense Fab packing and extensive Fab-Fab interactions, enabling the formation of larger assemblies. In contrast, Fab 7118 lacks stable homotypic Fab-Fab contacts, resulting in more independent, non-cooperative binding events that limit assembly size and reduce long-range structural order on rsCSP (S10 Fig). Importantly, despite this difference in assembly size, both 7118 and 311 exhibit high affinity and protective activity, indicating that maximal occupancy of the repeat region is not required for potent function. These findings underscore the significance of structural diversity within antibody lineages and highlight the critical role of CDRH3 in governing homotypic interactions. The presence or absence of Fab-Fab contacts and their contribution to overall binding avidity and neutralization may be influenced by subtle sequence differences within CDRs, even among antibodies derived from the same germline genes.

Notably, the head-to-head inter-Fab arrangement observed in Fab 7160 and 399 complexes differs from the previously characterized PfCSP-targeting antibodies, derived from the V_H_3-33 germline, which adopt helical or spiral arrangements stabilized by homotypic Fab-Fab contacts [28]. Head-to-head inter-Fab interactions have also been reported for V_H_3-33 Fab 1210 and V_H_3-23 Fab 1410 in complex with NANP_5_ [27]. However, in these cases, the homotypic interactions are asymmetric and mediated by somatically mutated residues, in contrast to the symmetric, predominantly germline-encoded contacts seen in Fab 7160 and Fab 399. This distinction supports the classification of 7160 and 399 mAbs as a distinct germline-encoded homotypic class of anti-CSP antibodies and reveals that some antibodies have an inherent capability in their germline gene sequences to form homotypic Fab-Fab interactions with minimal SHM. This self-association may be an efficient, built-in strategy that helps the immune system respond more effectively to highly repetitive targets, such as found in the CSP of malaria parasites. Overall, our study suggests that different germline-encoded strategies have evolved to engage the repetitive PfCSP epitope, with potential implications for B cell activation, antigen presentation, and the durability of antibody responses, with important implications for vaccine design and efficacy.

From a functional perspective, the ability of all three mAbs-399, 7160, and 7118 to inhibit liver-stage parasite burden by over 90% [20,25] indicates that both homotypic and non-homotypic modes of binding can provide strong protection. This finding suggests that, while homotypic Fab-Fab interactions may enhance antibody avidity and stability, they are not strictly required for protective efficacy if other factors can compensate. Our data suggest that CSP-targeting antibodies can achieve high avidity and protective efficacy through multiple strategies, either via multivalent epitope engagement stabilized by Fab-Fab interactions or through more extensive Fab-epitope interactions in the absence of homotypic contacts. In conclusion, our study explains how antibodies of the V_H_3-49/V_K_2D-29 lineage can achieve high-avidity binding to the PfCSP central repeat region through different binding mechanisms. These findings have several implications for malaria vaccine design. First, the identification of germline-encoded features that promote homotypic interactions, as in 7160 and 399, provides a potential target for germline-focused vaccine design aimed at eliciting antibodies with inherent capacity for cooperative binding and enhanced avidity. Second, the existence of multiple binding modes achieving similar potency suggests that immunogens can be designed to accommodate structurally diverse B-cell receptors within the same lineage, potentially broadening the range of antibodies elicited. Third, a detailed understanding of how CDRH3 length and Fab-Fab contacts influence binding can guide the engineering of immunogens that stabilize favorable antibody configurations and improve the strength and longevity of the antibody response. Overall, these data highlight that effective vaccines can elicit protective antibodies via multiple structural pathways. Structural insights from these distinct binding modes provide a rational framework for immunogen design, engaging diverse B-cell clones while maximizing avidity and protective efficacy, thereby providing further structural models for next-generation vaccines for malaria and other pathogens displaying repetitive epitopes.

## Methods and materials

### Protein expression and purification

The variable region sequences of the heavy and light chains for antibodies 7160 and 7118 were obtained from a published study by Atreca [20]. Fab genes were codon-optimized for mammalian expression and cloned into the PHCMV3 expression vector (GenScript). Transient transfection was performed in ExpiCHO cells to express Fabs 7160, 7118, and 399. Purification was carried out using a HiTrap Protein G HP column (GE Healthcare), followed by size-exclusion chromatography on a Superdex 200 16/90 column (GE Healthcare) in 1 × Tris-buffered saline (TBS; 50 mM Tris, pH 7.8, 140 mM NaCl).

### Biolayer Interferometry (BLI) assay for Fab binding kinetics

The binding kinetics of Fabs 399, 7160, and 7118 to various epitopes of PfCSP were evaluated using biolayer interferometry (BLI) on an Octet Red system (Pall ForteBio). Biotinylated peptides were synthesized at Innopep Inc. and diluted to 10 μg/mL in kinetics buffer (1 × PBS supplemented with 0.01% BSA and 0.002% Tween 20). Peptides were immobilized on streptavidin-coated biosensors by incubating for 600 s, followed by a 40 s baseline equilibration in kinetics buffer. For the association, Fabs were serially diluted for major repeat peptides (200, 100, 50, 25, 12.5, and 6.25 nM) and for minor and junctional peptides (800, 400, 200, 100, 50, and 25). The binding to recombinant CSP (rsCSP) was assessed similarly. Biotinylated-rsCSP was loaded onto streptavidin biosensors (Pall ForteBio, cat No 18-5019) at 10 μg/mL in kinetics buffer. Sensors were then dipped into Fab solutions at concentrations of 200, 100, 50, 25, 12.5, 6.25, and 3.15 nM, respectively. The assay workflow consisted of the following steps: 1) baseline in kinetics buffer for 60 s; 2) antigen loading for 60 s; 3) baseline for 60 s; 4) association for 300 s; and 5) dissociation in kinetics buffer for 300 s. Background subtraction was performed using reference sensors in buffer alone to assess non-specific binding. Kinetic parameters were derived using the Octet data analysis software (version 12.2), with data fitted to a 1:1 binding model.

All measurements were performed in at least two independent experiments with similar results. Representative sensorgrams are shown, and fitted parameters are reported. Goodness-of-fit was assessed based on R² values.

### Fab-peptide complex formation

All CSP-derived peptides used in this study were N- and C-terminally protected with acetyl and amide groups, respectively. The sequences were as follows: Ac-NPDPNANPNVDPNANP-NH₂ (junctional region peptide), Ac-NVDPNANPNVDPNANPNVDP-NH₂ (minor repeat region peptide), Ac-NPNANPNANPNA-NH₂ (short major repeat region peptide), and Ac-NANPNANPNANPNANPNANPNANP-NH₂ (long major repeat region peptide). All peptides were synthesized by Innopep Inc. at >98% purity. Fab 7160 was concentrated to 10 mg/mL in 1X TBS (Tris-buffered saline) using 10K Amicon ultra centrifugal filters and incubated with the junctional, minor repeat, and major repeat peptides at a 1:5 Fab-to-peptide molar ratio. Similarly, Fab 7118 was concentrated to 10 mg/mL in 1X TBS and incubated with the minor repeat and long major repeat (NANP₆) peptides at a 1:5 molar ratio. All Fab-peptide mixtures were stored at 4 °C overnight.

### Crystallization of Fabs and complexes

All crystallization screening was performed using our high-throughput robotic CrystalMation system (Rigaku) at The Scripps Research Institute (TSRI). Crystals were obtained using the sitting-drop vapor diffusion method at 293 K, with reservoir solutions of 35 µL and drops consisting of 0.1 µL of protein mixed with 0.1 µL of precipitant solution.

Crystals of unliganded Fab 399 were grown in 0.2 M potassium formate and 20% (w/v) polyethylene glycol (PEG) 3350, whereas crystals of Fab 7160 were obtained in 0.1 M HEPES (pH 7.5) and 20% (w/v) PEG 8000. Crystals of Fab 7160 in complex with the junctional region peptide were grown in 0.2 M sodium dihydrogen phosphate (pH 4.5) and 20% (w/v) polyethylene glycol (PEG) 3350. The Fab 7160-minor repeat peptide complex crystallized in 0.1 M HEPES (pH 7.0), 1 M lithium chloride, and 20% (w/v) PEG 6000. Fab 7160 co-crystallized with the NPNA₃ peptide in 0.2 M sodium acetate (pH 7.0) and 20% (w/v) PEG 3350. Crystals of the Fab7160-NANP₆ complex were grown in 0.2 M magnesium nitrate (pH 5.8) and 20% (w/v) PEG 3350. All crystals in this group were cryoprotected using 10% ethylene glycol before data collection.

For Fab 7118 complexes, co-crystals with the minor repeat peptide were obtained in 0.1 M HEPES (pH 7.5), 30% (v/v) 1,2-propanediol, and 20% (v/v) PEG 400. Crystals of the Fab 7118-NANP₆ complex were grown in 0.1 M sodium cacodylate (pH 6.5), 0.16 M calcium acetate, 14.4% (w/v) PEG 8000, and 20% (v/v) glycerol. Crystals of unliganded Fab 7118 were grown in 0.2 M potassium formate, 20% (w/v) PEG 3350, and 10% (v/v) ethylene glycol. Crystals for both Fab 7160 and Fab 7118, unliganded and complexes, typically appeared within 14–21 days at 293 K.

Unliganded Fab 7160 and Fab 399 crystallized in the P2₁ space group, each containing two Fab molecules in the asymmetric unit (ASU). Fab 7160 in complex with the junctional peptide crystallized in the P2₁2₁2₁ space group with eight Fab-peptide complexes in the ASU. The Fab 7160 complex with the minor repeat peptide also crystallized in P2₁2₁2₁ but contained two Fab-peptide complexes per ASU. The Fab 7160 complex with the short major repeat peptide crystallized in the C2_1_ space group with two Fab-peptide complexes in the ASU, whereas the complex with the long major repeat peptide crystallized in P2₁ with two complexes per ASU. For comparison, crystals of unliganded Fab 7118 and Fab 7118 in complex with the minor and major repeat peptides crystallized in the P2₁2₁2₁ space group, each containing a single Fab molecule or Fab-peptide complex in the ASU.

### X-ray data collection and structure determination

X-ray diffraction data for unliganded Fab 7160 and Fab 7160 in complex with the junctional and minor repeat peptides were collected at the Advanced Light Source (ALS) beamline BL 5.0.2. Data for Fab 7160 in complex with the short (NPNA₃) and long major (NANP₆) repeat peptides were collected at beamline 17-ID-1 (AMX) at the National Synchrotron Light Source II (NSLS-II). Diffraction data for unliganded Fabs 399 and 7118, as well as Fab 7118 in complex with the minor repeat and long major repeat peptides, were collected at Stanford Synchrotron Radiation Lightsource (SSRL) beamline BL 12–1.

All datasets were processed and scaled using the HKL-2000 package [32]. The structures of Fabs 7160 and 7118 were determined by molecular replacement using PHASER [33] with homology models generated by AlphaFold2 [34]. Model building was performed in Coot, and structural refinement was carried out using phenix.refine [35,36]. Peptides were manually fitted into the Fo–Fc electron density maps, followed by multiple cycles of refinement in phenix.refine [35]. Fab residue numbering follows the Kabat nomenclature. Structure validation was performed using MolProbity [37]. Buried surface areas were calculated with MS [38], and hydrogen bonds were analyzed using HBPLUS [39].

### Cryo-EM sample preparation and data collection

To prepare complexes for cryo-EM, 166 µg of Fab 7118 or Fab 7160 was mixed with 10 µg of rsCSP and incubated overnight at 4°C, followed by purification of the complex by size-exclusion chromatography on a Superdex 200 10/300 column (GE Healthcare), concentrated to around 1 mg/mL, and applied on a glow-discharged Quantifoil 1.2/1.3 300-mesh copper grid (Quantifoil). Grids were blotted for 5 sec after a 10 sec wait time in a Vitrobot Mark IV (ThermoFisher) at 4°C and 100% humidity before being plunged in liquid ethane.

Movies were collected on a Falcon 4i detector (ThermoFisher) mounted to a Glacios 2 microscope (ThermoFisher) operating at 200 keV, with nominal magnification of 190,000X and pixel size of 0.718 Å. Data collection was performed through the EPU interface (ThermoFisher), at an expected dose rate of 60 e^-^/Å^2^ and defocus range between -0.6 µm and -1.6 µm, for a total of 2065 movies (Fab 7118) or 2541 (Fab 7160).

### Cryo-EM data processing and model building

CryoSPARC [40] and CryoSPARC Live were used for data processing. Patch Motion Correction and CTF estimation were performed by cryoSPARC Live, and movies with a CTF fit higher than 10 Å were rejected from further processing. An initial batch of particles was picked with Blob Picker (minimal diameter 140 Å), resulting after a first round of 2D classification in a set of templates used for Template Picking on all micrographs. After 2D classification and ab-initio model building, a first set of 250,774 particles was selected for Fab 7118 and 289,602 particles for Fab 7160, at which point the ab-initio job had isolated dimeric Fab 7160 from higher-order oligomers. The higher-order oligomers were processed independently but suffered from too much orientation bias and could not be brought to high-resolution. For Fab 7118, the best ab-initio class was first processed through a Non-Uniform Refinement (NU-R) job, after which Global CTF Refinement was applied before another NU-R to obtain the final map. For Fab 7160, the particles were brought to a last round of high-resolution ab-initio building (maximum resolution 3 Å), homogeneous reconstruction and local refinement (static mask), following recent published protocols [41], to obtain the final map, with 116,764 particles (Fab 7160). The set of 250,774 particles for Fab 7118 that led to the final map was also used for the 3DVA job, using default parameters (3 modes, C1, 20 iterations, lambda 0.01) and a filtered resolution of 6 Å. For model building, the cryo-EM maps, after checking for handedness, were used as inputs to generate Fab fragments with ModelAngelo [42]. The models were then ported to Coot [36], to build CSP peptides, complete the Fab molecules, and were then refined using Phenix [35], iterated with Coot if necessary until Ramachandran and geometry parameters were within allowed constraints, and finally validated with MolProbity [37].

### Molecular dynamics simulation

We used the cryo-EM structures of the antibodies 311 (PDB: 8DYX) and 7118 (this paper) in complex with rsCSP as starting structures for molecular dynamics simulations (MD). Starting structures for MD simulations were prepared in Molecular Operating Environment (Chemical Computing Group, version 2024.06 using the Protonate3D tool [43]. To neutralize the charges, the uniform background charge was applied, which is required to compute long-range electrostatic interactions. [44,45]. Using the tleap tool of the AmberTools24 package, the structures were soaked in cubic water boxes of TIP3P water molecules with a minimum wall distance of 12 Å to the protein [46,47]. For all simulations, parameters of the AMBER force field 14SB were used [48]. We then performed three repetitions of each 1 µs of classical molecular dynamics simulations for each complex using Amber24 [46,47]. MD simulations were performed in an NpT ensemble using pmemd.cuda [49]. Bonds involving hydrogen atoms were restrained by applying the SHAKE algorithm, allowing a time step of 2 fs [50]. The Langevin thermostat was used to maintain the temperature during simulations at 300 K with a collision frequency of 2 ps^−1^ and a Monte Carlo barostat with one volume change attempt per 100 steps [51–53].

The interaction energies were calculated with CPPTRAJ using the interaction energy (LIE) tool [54]. The electrostatic interaction energies were calculated for all frames of each simulation and provided the simulation-averages of these interactions. To calculate the interactions and interaction frequencies of the binding interface, we used the GetContacts tool [55]. Cluster analysis was performed in CPPTRAJ, aligning and clustering on all Cα atoms using a distance cut-off criterion of 4 Å. PyMOL was used for visualization of the clusters and the interaction networks (The PyMOL Molecular Graphics System, Version 3.0 Schrödinger, LLC.) [30].

## Supporting information

S1 Fig Homotypic Fab-Fab interactions of unliganded Fabs 7160 and 399 and buried surface area (BSA) contributions of Fab 7160 CDRs to different PfCSP regions. A) Structural details of homotypic Fab-Fab interactions observed for unliganded Fab 7160 and Fab 399. Two interacting Fab molecules are shown for each antibody. Fab A is colored grey and blue for Fab 7160 and Fab 399, respectively. Fab B is colored pink for both antibodies. Black dashed lines indicate hydrogen bonds at the Fab-Fab interface. Fabs are shown as cartoon representations, with side chains of interacting residues displayed as sticks. B) The bar plot shows the relative BSA contributed by Fab 7160 CDRs H2, H3, L1, and L3 in complexes with the junctional region (magenta), minor repeat region (blue), and major repeat region (salmon). The buried surface area (BSA) contributed by each CDR to the Fab7160-peptide interaction is shown in the table.
(TIF)

S2 Fig Structural analysis of CSP peptides bound to 7160 Fab. The structures of the junctional (magenta), minor repeat (cyan), short major repeat (salmon), and long major repeat (yellow) peptides in the complexes with 7160 Fab are represented as sticks. Type I β-turns (black circle) and Asn pseudo 3_10_ turns (red circle) in the bound peptides are highlighted. Hydrogen bonds are shown as black dashes. The lower panel shows the Ramachandran plots [56] for the dihedral angles of the various peptides bound to 7160 Fab.
(TIF)

S3 Fig Hydrogen-bond interactions of Fabs 7160 and 399 with NANP_6_ peptide and analysis of Fab residues in the homotypic Fab-Fab interface. **(A-B)** Hydrogen-bond interactions between Fab 7160 (grey) and Fab 399 (blue) with NANP_6_ (yellow). Contacts between the two Fabs (Fab A and Fab B) in the homotypic Fab-Fab interaction, which simultaneously recognize the peptide, are highlighted. **(C-D)** A bar plot displays individual residue contributions to the buried surface area (BSA) at the homotypic Fab-Fab interface for the heavy and light chains of Fab 7160 (grey) and Fab 399 (blue). CDRs are highlighted in red in Kabat designation. Additionally, sequence alignment of the heavy and light chains with the germline gene is shown to identify somatically mutated residues. Residues involved in hydrogen bonds and salt bridges are annotated with “H” and “S,” respectively.
(TIF)

S4 Fig Cryo-EM of mAb 7160 in complex with rsCSP. **(A)** Representative micrographs at ~1.3 um defocus of the complexes with no filter (left) and a low-pass filter of 6 Å. **(B)** Representative 2D classes of the complex. **(C)** GS-FSC and cFAR FSC of the complex map used in the reconstruction of the model. **(D)** Local resolution estimates of the map used in the reconstruction, displayed at two different points of view, with a color-coded legend indicating local resolution in Å.
(TIF)

S5 Fig Comparison of 7160 cryo-EM and X-ray structures, and higher order oligomerization of 7160. **(A)** Side-by-side comparison of the x-ray structure of Fab 7160 bound to NANP_6_ with the corresponding region of the cryo-EM structure of the 7160–rsCSP complex. Two Fabs that form homotypic interactions and contact the peptide are shown in pink (Fab A) and grey (Fab B). The CSP-derived NANP_6_ peptide is shown in yellow and rsCSP in cyan, and represented as tubes (top) and as sticks (below). The N and C-termini of the peptides shown are indicated. **(B)** Representative 2D class (left) and 3D map (right) of higher-order oligomers averages observed by cryo-EM single-particle analysis. The averaged particle images, grouped during data processing, illustrate a population of two Fab 7160 dimers binding to the same rsCSP and resulting in dimer-of-dimer complexes. The head-to-head interfaces are observed in proximity to one another in the center of the 2D classes, with the C_H_1 constant domains for each Fab pointing outwards away from the complexes. The lack of alternative views in the cryo-EM data precluded the generation of higher-resolution 3D models from these particles, leading to orientation bias reflected in the cryo-EM map shown.
(TIF)

S6 Fig Structural comparison of Fabs 399, 7160, and 7118 highlights distinct recognition of NANP peptides. **(A)** CDRH3 regions of Fab 399 (blue), 7160 (grey), and Fab 7118 (salmon) are shown in stick representation. **(B-C)** Superposition of CDRH3 regions of Fab 399, 7160, and 7118 indicates that the elongated CDRH3 of Fab 7118 may interfere with Fab-Fab homotypic interactions. **(D)** Structural alignment of Fab-NANP₆ complexes reveals distinct peptide binding modes for Fab 7160 (yellow) and Fab 7118 (magenta). The N- and C-termini of the bound peptides are indicated.
(TIF)

S7 Fig Cryo-EM of mAb 7118 in complex with rsCSP. **(A)** Representative micrographs at ~1.3 um defocus of the complexes, with no filter (left) and a low-pass filter of 6 Å. **(B)** Representative 2D classes of the complex. **(C)** GS-FSC and cFAR FSC of the complex map used in the reconstruction of the model. **(D)** Local resolution estimates of the map used in the reconstruction, displayed at two different points of view, with color coded legend indicating local resolution in Å.
(TIF)

S8 Fig Structural comparison of Fab 7118 with Fab 7160 and Fab 399 binding. Superposition of Fab variable regions from two molecules of 7118-rsCSP cryo-EM structure (Fab A: green, Fab B: pink) with two molecules of Fab 7160-NANP_6_ crystal structure (grey) in the upper panel and two molecules of Fab 399-NPNA_6_ crystal structure (blue) shown in the lower panel. Models were aligned using PyMOL. Cartoon representation of the alignment between two 7118-rsCSP Fv molecules and the 7160-NANP_6_ complex. Asp^99^ in the CDRH3 region in 7118 is highlighted and represented as a stick. This residue would appear to introduce steric hindrance in 7118 at the inter-Fab interface, disfavoring the symmetric Fab-Fab interactions that are observed in the Fab 7160 and 399 complexes.
(TIF)

S9 Fig 3D variability analysis from the cryo-EM of 7118-rsCSP complex. **(A)** Orthogonal slices for all three eigenvectors solved during 3D Variability Analysis of the final particles set, with changes in voxel value colored from red (positive) to blue (negative). **(B)** Coordinates distribution of the 3DVA, confirming the homogeneity of the motion observed.
(TIF)

S10 Fig Distinct quaternary binding architectures and inter-Fab interactions of CSP-targeting antibodies. **(A)** Left, Fab 399 (blue) adopts a head-to-head, symmetric arrangement with inter-Fab interactions primarily mediated by germline-encoded residues. The middle panel shows Fab 7118 (wheat), when bound to rsCSP, forms a quaternary spiral architecture but lacks homotypic inter-Fab contacts or shows transient contacts. The right panel shows Fab 311 (cyan) binds rsCSP with ~11 Fabs forming a spiral assembly stabilized by inter-Fab interfaces enriched in somatically mutated residues. **(B)** The inter-Fab contacts emphasize how 7118 exhibits a hybrid structural feature between the more extensive homotypic interactions observed for 311 and 399.
(TIF)

S1 Movie 3D variability analysis of Fab-CSP spiral architecture. 3D Variability Analysis (3DVA) performed on particles from the final cryo-EM reconstruction. The movie shows a homogeneous spiral architecture in the absence of apparent homotypic contacts. Most of the conformational variability corresponds to individual Fab molecules pivoting about a more constrained CSP spiral.
(MP4)

S1 Table Biolayer interferometry kinetics of Fab binding to CSP-derived peptides and recombinant-shortened CSP (rsCSP). (DOCX)

S2 Table X-ray data collection and refinement statistics for unliganded Fabs 399, 7160, and 7118. (DOCX)

S3 Table X-ray data collection and refinement statistics for 7160 Fab with CSP-derived peptides. (DOCX)

S4 Table X-ray data collection and refinement statistics for 7118 Fab with CSP-derived peptides. (DOCX)

S5 Table A. Hydrogen bonds between Fab 7160 and (NANP)_6_ peptide. B. Hydrogen bonds between Fab 7118 and (NANP)_6_ peptide.
(DOCX)

S6 Table Cryo-EM structure data collection and refinement statistics of Fabs 7118 and 7160 with rsCSP. (DOCX)

S7 Table Mean electrostatic energy of 7118 Fab-rsCSP interfaces calculated during molecular dynamics simulations. (DOCX)

S8 Table Genetic features and clonality of CSP-binding antibodies. (DOCX)
