# Supplementary material for: Structural basis for conserved and distinct antigen recognition by a lineage of malaria-protective antibodies
Source: PLoS Pathog. 2026 Jun 3;22(6):e1014243. doi: 10.1371/journal.ppat.1014243 (PMC13249157; doi:10.1371/journal.ppat.1014243)
Supplement: S1 Table — (DOCX) [file ppat.1014243.s012.docx]

**S1 Table. Biolayer interferometry kinetics of Fab binding to CSP-derived peptides and recombinant-shortened CSP (rsCSP)**

| **Fab** | **K_D_ (M)** | **K_D_ Error** | **k_on_ (1/Ms)** | **k_on_ Error** | **k_off_ (1/s)** | **k_off_ Error** |
| --- | --- | --- | --- | --- | --- | --- |
| **NPDPNANPNVDPNANP (Junctional region)** | | | | | | |
| 399  Replicate | 2.04E-07  1.91E-07 | 5.65E-10  5.64E-10 | 7.19E+03  9.14E+03 | 1.45E+01  2.07E+01 | 1.47E-03  1.74E-03 | 2.77E-06  3.30E-06 |
| 7160  Replicate | 7.01E-07  6.62E-07 | 1.45E-09  1.73E-09 | 8.55E+03  8.95E+03 | 1.66E+01  2.18E+01 | 5.99E-03  5.93E-03 | 4.25E-06  5.49E-06 |
| 7118  Replicate | 6.12E-07  6.43E-07 | 3.21E-09  2.26E-09 | 2.86E+04  2.47E+04 | 1.46E+02  8.29E+01 | 1.75E-02  1.58E-02 | 1.97E-05  1.69E-05 |
| **NVDPNANPNVDPNANPNVDP (Minor repeat region)** | | | | | | |
| 399  Replicate | 2.42E-07  2.47E-07 | 6.07E-10  6.41E-10 | 1.04E+04  1.23E+04 | 2.27E+01  2.82E+01 | 2.53E-03  3.06E-03 | 3.17E-06  3.76E-06 |
| 7160  Replicate | 7.48E-07  7.03E-07 | 1.75E-09  1.92E-09 | 1.22E+04  1.81E+04 | 2.72E+01  3.04E+01 | 9.16E-03  8.31E-03 | 6.75E-06  7.43E-06 |
| 7118  Replicate | 4.84E-08  3.55E-08 | 1.14E-10  9.24E-11 | 3.15E+04  9.49E+04 | 5.82E+01  2.25E+02 | 1.53E-03  3.37E-03 | 2.27E-06  3.59E-06 |
| **NPNA_3_ (Short major repeat region)** | | | | | | |
| 399  Replicate | 2.57E-08  2.70E-08 | 7.64E-11  1.97E-11 | 3.29E+04  1.43E+04 | 5.29E+01  3.08E+01 | 8.85E-04  3.87E-04 | 2.12E-06  2.70E-06 |
| 7160  Replicate | 2.06E-08  1.96E-08 | 9.34E-11  9.96E-11 | 2.34E+04  2.34E+04 | 3.79E+01  4.07E+01 | 4.85E-04  4.59E-04 | 2.04E-06  2.19E-06 |
| 7118  Replicate | 1.07E-08  1.07E-08 | 3.87E-11  4.17E-11 | 4.55E+04  1.27E+04 | 6.52E+01  3.07E+02 | 4.89E-04  1.36E-04 | 1.62E-06  3.55E-06 |
| **NANP_6_ (Long major repeat region)** | | | | | | |
| 399  Replicate | 1.06E-08  1.15E-08 | 3.98E-11  5.25E-11 | 8.61E+04  5.77E+04 | 1.94E+01  1.24E+02 | 9.16E-04  6.64E-04 | 2.74E-06  2.67E-06 |
| 7160  Replicate | 8.03E-09  9.08E-09 | 2.20E-11  4.41E-11 | 8.26E+04  5.84E+04 | 1.14E+02  1.31E+02 | 6.64E-04  5.30E-04 | 1.60E-06  2.36E-06 |
| 7118  Replicate | 5.71E-09  6.72E-09 | 1.51E-11  3.44E-11 | 8.89E+04  6.78E+04 | 9.57E+01  1.27E+02 | 5.08E-04  4.56E-04 | 1.23E-06  2.17E-06 |
| **rsCSP (recombinant shortened CSP)** | | | | | | |
| 399  Replicate | 2.93E-09  2.93E-09 | 4.08E-11  3.89E-11 | 7.64E+04  7.84E+04 | 1.98E+02  1.94E+02 | 2.23E-04  2.26E-04 | 3.07E-06  3.00E-06 |
| 7160  Replicate | 3.44E-09  2.93E-09 | 3.52E-11  3.33E-11 | 7.48E+04  7.55E+04 | 1.64E+02  1.59E+02 | 2.58E-04  2.21E-04 | 2.57E-06  2.41E-06 |
| 7118  Replicate | 5.28E-09  4.92E-09 | 1.81E-11  2.01E-11 | 6.72E+04  6.67E+04 | 6.84E+01  7.50E+01 | 3.55E-04  3.28E-04 | 1.16E-06  1.29E-06 |
