# Supplementary material for: Structural basis for conserved and distinct antigen recognition by a lineage of malaria-protective antibodies
Source: PLoS Pathog. 2026 Jun 3;22(6):e1014243. doi: 10.1371/journal.ppat.1014243 (PMC13249157; doi:10.1371/journal.ppat.1014243)
Supplement: S2 Table — (DOCX) [file ppat.1014243.s013.docx]

**S2 Table. X-ray data collection and refinement statistics for unliganded Fabs 399, 7160 and 7118**

|  | **399 unliganded** | **7160 unliganded** | **7118 unliganded** |
| --- | --- | --- | --- |
| **Data collection** | | | |
| Beamline | SSRL 12-1 | NSLS-II AMX | SSRL 12-1 |
| Wavelength (Å) | 0.97946 | 0.92010 | 0.97946 |
| Resolution (Å) | 50.00-2.29 (2.34-2.29)^a^ | 50.00-2.39 (2.44-2.39)^a^ | 50.00-2.29 (2.34-2.29)^a^ |
| Space group | P2_1_ | P2_1_ | P2_1_2_1_2_1_ |
| Unit cell a, b, c (Å) | 63.90, 86.93, 89.76 | 62.54, 85.23, 89.55 | 44.66, 73.20, 150.50 |
| α, β, γ (°) | 90, 100.82, 90 | 90, 101.23, 90 | 90, 90, 90 |
| Unique reflections | 41,653 (2,109)^a^ | 36,453 (1,800)^a^ | 21,869 (1,129) |
| Redundancy | 5.9 (6.5)^a^ | 6.8 (5.3)^a^ | 11.7 (12.9)^a^ |
| Completeness (%) | 97.0 (98.5)^a^ | 99.7 (99.9)^a^ | 94.7 (99.9)^a^ |
| Mean I/sigma (σ_I_) | 39.9 (10.9)^a^ | 9.5 (1.5)^a^ | 43.9 (16.5)^a^ |
| R_sym_ (%)^b^ | 8.0 (32.4)^a^ | 13.4 (56.4)^a^ | 8.2 (19.4)^a^ |
| R_pim_ (%)^b^ | 3.7 (13.9)^a^ | 5.6 (26.7)^a^ | 2.6 (5.6)^a^ |
| CC_1/2_ (%)^c^ | 99.3 (95.2)^a^ | 98.9 (68.3)^a^ | 99.9 (99.8)^a^ |
| **Refinement statistics** | | | |
| Resolution (Å) | 39.32-2.29 | 40.65-2.39 | 41.38-2.29 |
| Reflections (work) | 41,630 | 35,997 | 21,825 |
| Reflections (test) | 2,000 | 2,000 | 2,000 |
| R_cryst_^d^ / R_free_^e^ (%) | 24.1/27.9 | 22.8/26.2 | 21.8/27.7 |
| **Number of atoms** |  |  |  |
| Fab | 6,592 | 6,629 | 3,293 |
| Peptide | 0 | 0 | 0 |
| Water | 144 | 77 | 108 |
| **Average B-value (Å^2^)** |  |  |  |
| Fab | 37 | 36 | 28 |
| Peptide | 0 | 0 | 0 |
| Water | 31 | 29 | 26 |
| Wilson B (Å^2^) | 32 | 36 | 24 |
| **RMSD from ideal geometry** |  |  |  |
| Bond angle (^o^) | 0.56 | 0.52 | 0.62 |
| Bond length (Å) | 0.002 | 0.005 | 0.003 |
| **Ramachandran statistics^f^** |  |  |  |
| Favored (%) | 98.12 | 98.71 | 97.87 |
| Allowed (%) | 1.88 | 1.29 | 2.13 |
| Outliers (%) | 0.00 | 0.00 | 0.00 |
| **PDB Code** | 12FE | 12FD | 12FC |

^a^ Numbers in parentheses refer to the highest resolution shell.

^b^ Rsym = Σhkl Σi | Ihkl,i - | / Σhkl Σi Ihkl,i and Rpim = Σhkl (1/(n-1))1/2 Σi | Ihkl,i - | / Σhkl Σi Ihkl,i, where Ihkl,i is the scaled intensity of the ith measurement of reflection h, k, l, is the average intensity for that reflection, and n is the redundancy.

^c^ CC1/2 = Pearson correlation coefficient between two random half datasets.

^d^ Rcryst = Σhkl | Fo - Fc | / Σhkl | Fo | x 100, where Fo and Fc are the observed and calculated structure factors, respectively.

^e^ Rfree was calculated as for Rcryst, but on a test set comprising 5% of the data excluded from refinement.

^f^ From MolProbity (36).
