# Supplementary material for: Structural basis for conserved and distinct antigen recognition by a lineage of malaria-protective antibodies
Source: PLoS Pathog. 2026 Jun 3;22(6):e1014243. doi: 10.1371/journal.ppat.1014243 (PMC13249157; doi:10.1371/journal.ppat.1014243)
Supplement: S3 Table — (DOCX) [file ppat.1014243.s014.docx]

**S3 Table. X-ray data collection and refinement statistics for 7160 Fab with CSP-derived peptides**

|  | **7160 + junctional region** | **7160 + Minor repeat region** | **7160 + Short major repeat region** | **7160 + Long major repeat region** |
| --- | --- | --- | --- | --- |
| **Data collection** | | | | |
| Beamline | ALS 5.0.2 | ALS 5.0.2 | NSLS-II AMX | NSLS-II AMX |
| Wavelength (Å) | 0.97946 | 0.97946 | 0.92010 | 0.92010 |
| Resolution (Å) | 30.00-2.30 (2.34-2.30)^a^ | 50.00-2.27 (2.31-2.27)^a^ | 27.00-1.88 (1.93-1.88)^a^ | 50.00-2.33 (2.39-2.33)^a^ |
| Space group | P2_1_2_1_2_1_ | P2_1_2_1_2_1_ | C2_1_ | P2_1_ |
| Unit cell a, b, c (Å) | 88.19, 105.13, 405.18 | 87.00, 101.04, 104.84 | 188.01, 52.35, 102.98 | 103.21, 73.13, 134.37 |
| α, β, γ (°) | 90, 90, 90 | 90, 90, 90 | 90, 110.1, 90 | 90, 102.2, 90 |
| Unique reflections | 168,500 (8,359)^a^ | 42,381 (2,085)^a^ | 75,561 (3,681)^a^ | 82,863 (4,058)^a^ |
| Redundancy | 12.0 (11.3)^a^ | 6.3 (6.1)^a^ | 6.2 (4.3)^a^ | 6.6 (6.3)^a^ |
| Completeness (%) | 99.9 (99.9)^a^ | 97.7 (98.0)^a^ | 99.7 (97.4)^a^ | 99.9 (99.1)^a^ |
| Mean I/sigma (σ_I_) | 15.1 (1.7)^a^ | 14.7 (2.2)^a^ | 12.0 (1.2)^a^ | 13.1 (2.8)^a^ |
| R_sym_ (%)^b^ | 18.8 (103.9)^a^ | 16.0 (59.3)^a^ | 14.0 (60.9)^a^ | 14.9 (44.8)^a^ |
| R_pim_ (%)^b^ | 5.6 (32.1)^a^ | 6.8 (25.7)^a^ | 6.1 (31.0)^a^ | 6.2 (18.6)^a^ |
| CC_1/2_ (%)^c^ | 98.9 (73.5)^a^ | 99.5 (89.1)^a^ | 99.1 (63.8)^a^ | 98.3 (88.6)^a^ |
| **Refinement statistics** | | | | |
| Resolution (Å) | 29.88-2.30 | 46.53-2.27 | 26.79-1.88 | 41.74-2.33 |
| Reflections (work) | 167,046 | 42,148 | 75,043 | 81,800 |
| Reflections (test) | 2,000 | 2,007 | 2,000 | 1,999 |
| R_cryst_^d^ / R_free_^e^ (%) | 22.8/27.4 | 25.1/28.8 | 20.7/24.5 | 20.6/23.6 |
| **Number of atoms** |  |  |  |  |
| Fab | 26,634 | 6,683 | 6,636 | 13,318 |
| Peptide | 718 | 160 | 168 | 316 |
| Water | 338 | 120 | 369 | 275 |
| **Average B-value (Å^2^)** |  |  |  |  |
| Fab | 43 | 37 | 31 | 33 |
| Peptide | 44 | 38 | 30 | 37 |
| Water | 36 | 37 | 32 | 31 |
| Wilson B (Å^2^) | 38 | 34 | 25 | 30 |
| **RMSD from ideal geometry** |  |  |  |  |
| Bond angle (^o^) | 0.55 | 0.54 | 0.91 | 0.53 |
| Bond length (Å) | 0.002 | 0.002 | 0.007 | 0.002 |
| **Ramachandran statistics^f^** |  |  |  |  |
| Favored (%) | 98.13 | 97.51 | 97.85 | 98.64 |
| Allowed (%) | 1.87 | 2.49 | 2.15 | 1.36 |
| Outliers (%) | 0.00 | 0.00 | 0.00 | 0.00 |
| **PDB Code** | 9ZM7 | 9ZM8 | 9ZM9 | 9ZMA |

^a^ Numbers in parentheses refer to the highest resolution shell.

^b^ Rsym = Σhkl Σi | Ihkl,i - | / Σhkl Σi Ihkl,i and Rpim = Σhkl (1/(n-1))1/2 Σi | Ihkl,i - | / Σhkl Σi Ihkl,i, where Ihkl,i is the scaled intensity of the ith measurement of reflection h, k, l, is the average intensity for that reflection, and n is the redundancy.

^c^ CC1/2 = Pearson correlation coefficient between two random half datasets.

^d^ Rcryst = Σhkl | Fo - Fc | / Σhkl | Fo | x 100, where Fo and Fc are the observed and calculated structure factors, respectively.

^e^ Rfree was calculated as for Rcryst, but on a test set comprising 5% of the data excluded from refinement.

^f^ From MolProbity (36).
