# Supplementary material for: Structural basis for conserved and distinct antigen recognition by a lineage of malaria-protective antibodies
Source: PLoS Pathog. 2026 Jun 3;22(6):e1014243. doi: 10.1371/journal.ppat.1014243 (PMC13249157; doi:10.1371/journal.ppat.1014243)
Supplement: S4 Table — (DOCX) [file ppat.1014243.s015.docx]

**S4 Table. X-ray data collection and refinement statistics for 7118 Fab with CSP-derived peptides**

|  | **7118 + Minor repeat region** | **7118 + Long major repeat region** |
| --- | --- | --- |
| **Data collection** | | |
| Beamline | SSRL 12-1 | SSRL 12-1 |
| Wavelength (Å) | 0.97946 | 0.97946 |
| Resolution (Å) | 50.00-2.09 (2.14-2.09)^a^ | 50.00-2.00 (2.03-2.00)^a^ |
| Space group | P2_1_2_1_2_1_ | P2_1_2_1_2_1_ |
| Unit cell a, b, c (Å) | 73.84, 83.03, 88.46 | 74.15, 83.39, 89.55 |
| α, β, γ (°) | 90, 90, 90 | 90, 90, 90 |
| Unique reflections | 30,491 (1,561)^a^ | 37,945 (1,871)^a^ |
| Redundancy | 5.1 (6.3)^a^ | 6.0 (6.1)^a^ |
| Completeness (%) | 93.7 (98.5)^a^ | 99.5 (99.7)^a^ |
| Mean I/sigma (σ_I_) | 29.0 (4.7)^a^ | 38.6 (7.4)^a^ |
| R_sym_ (%)^b^ | 8.8 (47.2)^a^ | 8.3 (34.0)^a^ |
| R_pim_ (%)^b^ | 4.7 (20.3)^a^ | 3.8 (15.3)^a^ |
| CC_1/2_ (%)^c^ | 99.9 (86.8)^a^ | 99.9 (94.1)^a^ |
| **Refinement statistics** | | |
| Resolution (Å) | 37.94-2.09 | 41.70-2.00 |
| Reflections (work) | 30,283 | 37,842 |
| Reflections (test) | 1,999 | 2,002 |
| R_cryst_^d^ / R_free_^e^ (%) | 20.6/24.2 | 18.0/21.7 |
| **Number of atoms** |  |  |
| Fab | 3,337 | 3,346 |
| Peptide | 138 | 140 |
| Water | 133 | 251 |
| **Average B-value (Å^2^)** |  |  |
| Fab | 28 | 23 |
| Peptide | 31 | 23 |
| Water | 30 | 29 |
| Wilson B (Å^2^) | 38 | 23 |
| **RMSD from ideal geometry** |  |  |
| Bond angle (^o^) | 0.59 | 0.90 |
| Bond length (Å) | 0.002 | 0.007 |
| **Ramachandran statistics^f^** |  |  |
| Favored (%) | 98.43 | 98.00 |
| Allowed (%) | 1.57 | 1.78 |
| Outliers (%) | 0.00 | 0.22 |
| **PDB Code** | 9ZMB | 9ZMC |

^a^ Numbers in parentheses refer to the highest resolution shell.

^b^ Rsym = Σhkl Σi | Ihkl,i - | / Σhkl Σi Ihkl,i and Rpim = Σhkl (1/(n-1))1/2 Σi | Ihkl,i - | / Σhkl Σi Ihkl,i, where Ihkl,i is the scaled intensity of the ith measurement of reflection h, k, l, is the average intensity for that reflection, and n is the redundancy.

^c^ CC1/2 = Pearson correlation coefficient between two random half datasets.

^d^ Rcryst = Σhkl | Fo - Fc | / Σhkl | Fo | x 100, where Fo and Fc are the observed and calculated structure factors, respectively.

^e^ Rfree was calculated as for Rcryst, but on a test set comprising 5% of the data excluded from refinement.

^f^ From MolProbity (36).
