# Supplementary material for: Structural basis for conserved and distinct antigen recognition by a lineage of malaria-protective antibodies
Source: PLoS Pathog. 2026 Jun 3;22(6):e1014243. doi: 10.1371/journal.ppat.1014243 (PMC13249157; doi:10.1371/journal.ppat.1014243)
Supplement: S5 Table — B. Hydrogen bonds between Fab 7118 and (NANP)6 peptide. (DOCX) [file ppat.1014243.s016.docx]

**Table S5. Hydrogen bonds between Fab and peptide.**

**A.** Hydrogen bonds between Fab 7160 and (NANP)_6_ peptide

|  |  |  |  |  |  |
| --- | --- | --- | --- | --- | --- |
| NANP_6_ (BSA Å²) | **Distance (Å)** | **7160-HC**  **Fab A** | **7160-LC**  **Fab A** | **7160-HC**  **Fab B** | **7160-LC**  **Fab B** |
| Ala2 (92) |  |  |  |  |  |
| Ala-O | 3.33 |  | Trp^96^-NE1 |  |  |
| Asn3 (34) |  |  |  |  |  |
| Asn-O  Asn-O | 2.90  3.10 | Arg^52^-NH2  Arg^52^-NE |  |  |  |
| Asn5 (124) |  |  |  |  |  |
| Asn-ND2 | 2.97 | Gly^96^-O |  |  |  |
| Asn-O | 2.83 |  |  | Tyr^53^-OH |  |
| Ala6 (17) |  |  |  |  |  |
| Ala-O | 3.06 | Arg^52^-NE |  |  |  |
| Pro12 (11) |  |  |  |  |  |
| Pro-O | 3.60 |  |  |  | Asn^27E^-ND2 |
| Asn13 (62) |  |  |  |  |  |
| Asn-ND2 | 3.20 |  |  |  | Asp^28^-OD2 |
| Ala14 (49) |  |  |  |  |  |
| Ala-N | 3.16 |  |  |  | Asp^27D^-OD1 |
| Asn15 (47) |  |  |  |  |  |
| Asn-ND2 | 2.85 |  |  |  | Asp^93^-OD1 |
| Asn-17 (45) |  |  |  |  |  |
| Asn-ND2 | 2.94 |  |  | Glu^58^-OE2 |  |
| Asn-O | 2.92 |  |  | Arg^52^-NH2 |  |
| Ala18 (68) |  |  |  |  |  |
| Ala18-O | 3.38 |  |  |  | Arg^91^-NE |
| Ala18-O | 3.24 |  |  |  | Trp^96^-NE1 |
| Asn19 (21) |  |  |  |  |  |
| Asn-OD1 | 3.32 |  |  |  | Arg^91^-NH1 |
| Asn-O | 2.99 |  |  | Arg^52^-NE |  |
| Asn-O | 3.21 |  |  | Arg^52^-NH2 |  |
| Asn21 (123) |  |  |  |  |  |
| Asn-O | 3.19 | Tyr^53^-OH |  |  |  |
| Asn-ND2 | 2.96 |  |  | Gly^96^-O |  |
| Asn-ND2 | 3.50 |  |  |  | Ser^100^-OG |

**B. Hydrogen bonds between Fab 7118 and (NANP)_6_ peptide**

|  |  |  |  |
| --- | --- | --- | --- |
| NANP_6_ (BSA Å²) | **Distance (Å)** | **7118-HC** | **7118-LC** |
| Asn5 (50) |  |  |  |
| Asn-O | 2.86 |  | Ser^27A^-OG |
| Asn7 (102) |  |  |  |
| Asn-N | 2.76 |  | Leu^27C^-O |
| Asn-ND2 | 3.33 |  | Glu^27^-OE1 |
| Asn-ND2 | 2.92 |  | Ser^27A^-O |
| Pro8 (21) |  |  |  |
| Pro-O | 2.75 |  | Arg^27E^-NH2 |
| Ala10 (51) |  |  |  |
| Ala-O | 2.77 |  | His^27D^-ND2 |
| Asn11 (50) |  |  |  |
| Asn-ND2 | 3.24 |  | Ile^92^-O |
| Asn-ND2 | 3.40 |  | Asp^93^-OD1 |
| Asn13 (48) |  |  |  |
| Asn-O | 2.99 | Arg^52^-NH2 |  |
| Asn-ND2 | 2.66 | Glu^58^-OE2 |  |
| Ala14 (60) |  |  |  |
| Ala-O | 3.25 |  | Trp^96^-NE1 |
| Asn15 (33) |  |  |  |
| Asn-O | 2.87 | Arg^52^-NE |  |
| Asn17 (118) |  |  |  |
| Asn-O | 3.30 | Asn^53^-ND2 |  |
| Asn-ND2 | 2.93 | Arg^96^-O |  |
| Asn-ND2 | 3.07 | Asn^98^-O |  |
| Asn-ND2 | 2.88 | Phe^100^-O |  |
