# Supplementary material for: Structural basis for conserved and distinct antigen recognition by a lineage of malaria-protective antibodies
Source: PLoS Pathog. 2026 Jun 3;22(6):e1014243. doi: 10.1371/journal.ppat.1014243 (PMC13249157; doi:10.1371/journal.ppat.1014243)
Supplement: S6 Table — (DOCX) [file ppat.1014243.s017.docx]

**S6 Table. Cryo-EM structure data collection and refinement statistics of Fabs 7118 and 7160 with rsCSP**

|  | **7118 -rsCSP**  **EMDB-74167** | **7160-rsCSP EMDB-74165** |
| --- | --- | --- |
| **Data collection and processing** | | |
| Magnification | 190,000x | 190,000x |
| Voltage (kV) | 200 | 200 |
| Electron exposure (e^–^/Å^2^) | 60 | 60 |
| Defocus range (μm) | -0.6 to -1.6 | -0.6 to -1.6 |
| Pixel size (Å) | 0.718 | 0.718 |
| Symmetry imposed | C1 | C1 |
| Final particle images (no.) | 250,774 | 116,764 |
| Map resolution (Å) | 3.32 | 3.55 |
| FSC threshold | 0.143 | 0.143 |
| **Refinement statistics** | | |
| Map sharpening *B* factor (Å^2^) | -108 | -93 |
| **Model composition** | | |
| Non-hydrogen atoms | 7576 | 6857 |
| Protein residues | 981 | 902 |
| ***Average B* values (Å^2^)** | | |
| Fab | 69 | 78 |
| CSP | 57 | 67 |
| **RMSD** | | |
| Bond angle (^o^) | 0.65 | 0.63 |
| Bond length (Å) | 0.005 | 0.004 |
| **Validation** | | |
| MolProbity score | 1.82 | 1.18 |
| Clashscore | 6.37 | 2.95 |
| Poor rotamers (%) | 0.00 | 0.00 |
| **Ramachandran statistics** | | |
| Favored (%) | 92.52 | 97.52 |
| Allowed (%) | 7.48 | 2.48 |
| Disallowed (%) | 0.00 | 0.00 |
| **PDB Code** | 9ZFZ | 9ZFY |
|  |  |  |
