# Supplementary material for: Structural basis for conserved and distinct antigen recognition by a lineage of malaria-protective antibodies
Source: PLoS Pathog. 2026 Jun 3;22(6):e1014243. doi: 10.1371/journal.ppat.1014243 (PMC13249157; doi:10.1371/journal.ppat.1014243)
Supplement: S7 Table — (DOCX) [file ppat.1014243.s018.docx]

**S7 Table. Mean electrostatic energy of 7118 Fab-rsCSP interfaces calculated during molecular dynamic simulations.**

| **Interface** | **ELE (kcal/mol, mean ± std)** |
| --- | --- |
| Fab A - CSP | -120.37 ± 27.41 |
| Fab B - CSP | -122.10 ± 45.08 |
| Fab C - CSP | -139.64 ± 43.97 |
| Fab D – CSP | -59.82 ± 24.98 |
| All Fabs - CSP | -441.92 ± 68.84 |
