# Supplementary material for: Structural basis for conserved and distinct antigen recognition by a lineage of malaria-protective antibodies
Source: PLoS Pathog. 2026 Jun 3;22(6):e1014243. doi: 10.1371/journal.ppat.1014243 (PMC13249157; doi:10.1371/journal.ppat.1014243)
Supplement: S8 Table — (DOCX) [file ppat.1014243.s019.docx]

**S8 Table. Genetic features and clonality of CSP-binding antibodies**

| **Antibody** | **Donor/**  **Vaccinee** | **Heavy Chain**  **V(D)J** | **Light**  **Chain**  **VJ** | **SHM**  **(H + L)** | **CDRH3 length (aa)** | **CDRL3 length (aa)** | **Clonal relationship** |
| --- | --- | --- | --- | --- | --- | --- | --- |
| 399 | 5 | IGHV3-49/  IGHD6-13/ IGHJ4 | IGKV2D-29/ IGKJ1 | 28 | VGVVIATAVY  10 aa | MQRIDLPWT  9 aa | Main  clone (clonally expanded) |
| 7160 | 5 | IGHV3-49/  IGHD6-13/ IGHJ4 | IGKV2D-29/ IGKJ1 | 20 | VGIEVSTAVY  10 aa | MQRIDLPWT  9 aa | Related  clone |
| 7118 | 3 | IGHV3-49/  IGHD3-3/ IGHJ6 | IGKV2D-29/ IGKJ1 | 23 | VRTNDFRDMDV  11 aa | MQTIDLPWT  9 aa | Independent  clone |

- SHM = Number of nucleotides divergent from germline in heavy plus light chains
- CDR lengths are in amino acids (aa).
- Independent clones indicate that antibodies derive from distinct B-cell clones, even when they share the same germline gene usage.
